# Exploration of icariin analog structure space reveals key features driving potent inhibition of human phosphodiesterase-5

**DOI:** 10.1371/journal.pone.0222803

**Published:** 2019-09-20

**Authors:** Yasmin Chau, Fu-Shuang Li, Olesya Levsh, Jing-Ke Weng

**Affiliations:** 1 Whitehead Institute for Biomedical Research, Cambridge, Massachusetts, United States of America; 2 Department of Biology, Massachusetts Institute of Technology, Cambridge, Massachusetts, United States of America; Universidade do Porto, Faculdade de Farmácia, PORTUGAL

## Abstract

The natural product icariin inhibits human phosphodiesterase-5 (PDE5) and represents a unique pharmacophore for treating erectile dysfunction, pulmonary hypertension, and other diseases. In this study, we explore the available icariin-derived chemical scaffolds through medicinal chemistry to develop novel icariin PDE5 inhibitors with improved potency and specificity. We synthesized six novel semi-synthetic icariin analogs as well as three naturally occurring icariin analogs, and characterized the structure-activity relationship in the context of human PDE5 inhibition using *in vitro* enzyme inhibition and kinetics assays and molecular modeling. Mammalian-cell-based assays and *in vitro* enzyme inhibition assays against human PDE6C further helped to identify the most potent and selective icariin analogs. Our results reveal the synergistic contribution of functional groups at the C3 and C7 positions of the icariin backbone towards PDE5 inhibition. Whereas a hydrophobic and flexible alkanol group at the C7 position is sufficient to enhance icariin analog potency, combining this group with a hydrophilic sugar group at the C3 position leads to further enhancement of potency and promotes specificity towards PDE5 versus PDE6C. In particular, compounds **3** and **7** exhibit *K*_*i*_ values of 0.036 ± 0.005 μM and 0.036 ± 0.007 μM towards PDE5 respectively, which are approaching those of commercial PDE5 inhibitors, and can effectively reduce GMP levels in cultured human BJ-hTERT cells. This study identifies novel icariin analogs as potent and selective PDE5 inhibitors poised to become lead compounds for further pharmaceutical development.

## Introduction

Cyclic nucleotide phosphodiesterases (PDEs) are key regulators of intracellular levels of the ubiquitous second messenger molecules cyclic guanosine monophosphate (cGMP) and cyclic adenosine monophosphate (cAMP) in eukaryotes [1,2]. cGMP and cAMP are biosynthesized from their corresponding nucleoside triphosphates (NTPs) by specific nucleotidyl cyclases in response to upstream signals, such as hormones or neurotransmitters, and modulate a variety of downstream processes, such as cell contractility, cell growth and proliferation, inflammation, sensory transduction, and neuronal plasticity [1,2]. PDEs turn off this signaling pathway by hydrolyzing cGMP and cAMP to produce the corresponding 5′-monophosphates GMP and AMP. Each of the 25 PDEs encoded by the human genome possesses characteristic substrate specificity and tissue expression patterning, and play distinct roles in normal physiology and diseases [2–4]. As a result, PDE inhibitors have long been developed as therapeutics with diverse disease indications [5].

Phosphodiesterase 5 (PDE5) is a cGMP-specific PDE expressed in all tissues, most prominently in smooth muscle cells, platelets, pancreatic cells, and cardiomyocytes [6]. PDE5 inhibitors are the most commercially successful PDE-targeting therapeutics. In particular, sildenafil (Viagra, Revatio; PubChem CID 135398744), vardenafil (Levitra; PubChem CID 135400189), and tadalafil (Cialis, Adcirca; PubChem CID 110635) are FDA-approved blockbuster drugs that treat erectile dysfunction and pulmonary hypertension. These drugs function as competitive inhibitors of PDE5 with IC_50_ values in the low nanomolar range (sildenafil, 1–9 nM; vardenafil, 0.1–0.8 nM; tadalafil, 1–7 nM) [6,7]. However, sildenafil and vardenafil also show inhibition of PDE6 isoforms, which are cGMP phosphodiesterases expressed in retina that play an important role in signaling of vision [8,9]. As a result, significant but transient visual disturbance is a common side effect associated with PDE5 inhibitors. In the case of on-demand treatment of erectile dysfunction, the three commercial PDE5 inhibitors exhibit a duration of action ranging from 3 hours to 17.5 hours [10,11], which may not be desirable for some users. Furthermore, recent studies suggest that PDE5 is a viable therapeutic target for the treatment of a wide range of illnesses, including cardiovascular, gastrointestinal, pulmonary, musculoskeletal, neurological, and reproductive diseases [12]. Alternative PDE5 inhibitors more specific for PDE5 and with different pharmacokinetic profiles will continue to be in demand.

**Fig 1:**
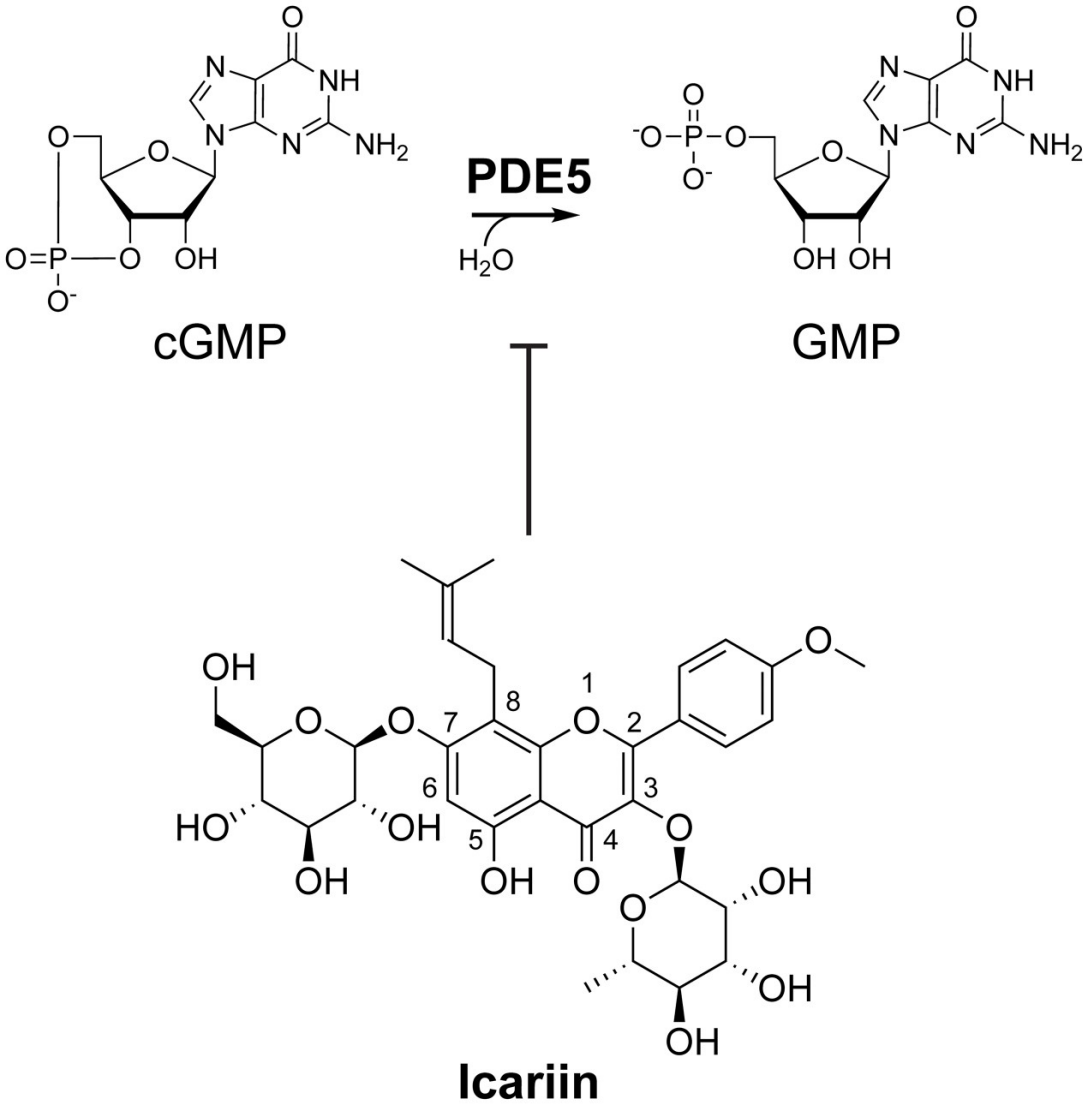
The natural product icariin inhibits phosphodiesterase-5 (PDE5). PDE5 hydrolyzes cGMP (cyclic guanosine monophosphate) to GMP (guanosine monophosphate). Icariin is a flavonoid known to competitively inhibit PDE5 with an IC_50_ around 1–6 μM [16–18].

Plants produce a plethora of structurally and functionally diverse secondary metabolites, many of which display bioactivities with potential utility in improving human health [13]. Identification of the natural products underlying the pharmacological activities of medicinal plants can reveal new compound classes that can be further developed into therapeutics. The horny goat weed, referring to several species belonging to the *Epimedium* genus, has been used for thousands of years as a traditional herbal medicine in China to treat sexual dysfunction, hypertension, rheumatic arthritis, and osteoporosis [14,15]. In a previous work, the prenylated flavonoid icariin (PubChem CID 5318997, Fig 1) and its naturally occurring analogs were identified as the principal bioactive components in *Epimedium* plants [14]. Icariin inhibits human PDE5 with an IC_50_ in the low- to mid-micromolar range (1–6 μM) [16–18]. Like several synthetic PDE5 inhibitors, icariin treatment can also increase cGMP levels in cavernous smooth muscle cells isolated from mice [17,19], making it a promising pharmacophore for developing new PDE5 inhibitors. In a previous study, Dell’Agli et al. synthesized three novel and two naturally occurring icariin derivatives that exhibit potency against PDE5 [18]. However, additional promising icariin structure space has not been fully explored, limiting our understanding of the structure-activity relationship of PDE5 inhibition by icariin analogs.

In this study, we aim to expand the chemical space of icariin-derived scaffolds and identify new icariin-based PDE5 inhibitors with enhanced potency and specificity. We systematically characterize the structure-activity relationship for six novel semi-synthetic icariin analogs (**3–8**) together with three previously described and naturally occurring icariin analogs (compounds **1**, **2**, and **9**) in the context of human PDE5 inhibition. We report new icariin analogs with IC_50_ and *K*_*i*_ values approaching synthetic PDE5 inhibitors. Our lead compounds exhibit minimal effects on the mitochondrial viability of several human cell lines, display the ability to decrease cellular GMP concentrations, and maintain specificity for PDE5 over PDE6C.

## Results and discussion

### Design and synthesis of icariin analogs

**Fig 2:**
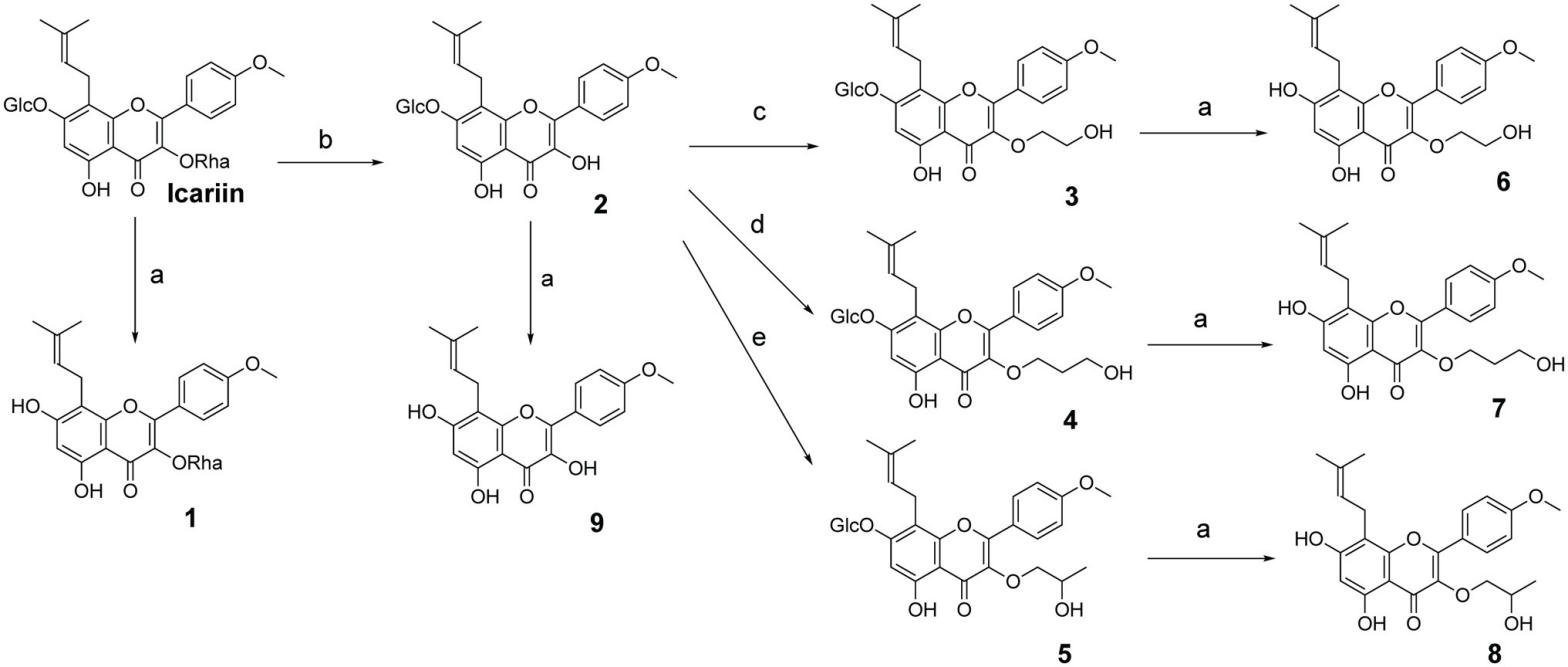
Structures and synthetic routes of icariin analogs 1–9. (A) Cellulase, 0.1 M NaOAc, pH = 5.7, 37 °C, overnight. (B) 5% H_2_SO_4_, 50 °C, 24h. (C) 2-bromoethanol, K_2_CO_3_, refluxed, 8h. (D) 3-Bromo-1-propanol, K_2_CO_3_, refluxed, 8h. (E) 1-bromo-2-propanol, K_2_CO_3_, 75°C, 6h. Glc = glucose, Rha = rhamnose. **5** and **8** are racemic mixtures.

Functional groups at the 3-*O* and 7-*O* positions of the icariin backbone seem poised to modulate PDE5 inhibition. A crystal structure of the human PDE5 catalytic domain bound to icariside II (**1** in Figs 2 and 4A) shows that the 3-*O*-rhamnose moiety is buried in a hydrophobic pocket of the active site [20]. Based on this structural information, we hypothesized that replacing the bulky and hydrophilic 3-*O*-rhamnose with a smaller, flexible, and more hydrophobic moiety might enhance icariin’s potency. The crystal structure also shows that the 7-OH of **1** is located near the active-site entrance and forms a hydrogen bond with Ser668 on the flexible H-loop (residues 660–683) [20]. Other crystal structures of PDE5 also show varying secondary structures of the H-loop upon binding to different inhibitors, even when the inhibitors share the same structural backbone (e.g., sildenafil and vardenafil) [20–23]. As a result, we hypothesized that retaining the icariin 7-*O*-glucose on our analogs may have minimal negative effects on PDE5 inhibition, since it is located at the active-site entrance near the conformationally flexible H-loop.

To test these hypotheses, we synthesized a series of nine icariin analogs (Fig 2, S1 to S9 Figs). First, the 7-*O*-glucose or the 3-*O*-rhamnose was replaced with a free hydroxyl group (**1** and **2** respectively) to evaluate the contribution of each sugar group to PDE5 inhibition. We then modified the 3-OH of compound **2** with a hydroxyethyl, hydroxypropyl, or hydroxyisopropyl group (compounds **3**, **4**, and **5** respectively). To evaluate the contribution of 3-*O*-alkanol to PDE5 inhibition independent of the 7-*O* position, we replaced the 7-*O*-glucose in compounds **3–5** with a free hydroxyl group to give compounds **6–8**. We also synthesized the aglycone of icariin (**9**) to determine the contribution of the core structure to PDE5 inhibition.

We evaluated icariin and all icariin analogs for characteristics of pan-assay interference compounds (PAINS) [24] (S10 Fig). PAINS molecules inhibit targets by non-specific mechanisms, usually by aggregation-induced inhibition [24]. Two assays were conducted: 1) non-specific inhibition of the enzyme trypsin, and 2) reduced inhibition of modest increases in PDE5 concentration. In the first assay, only compound **9** showed statistically significant inhibition of trypsin activity (p<0.05) with increasing concentrations of compound. In contrast, luteolin, a known small molecule aggregator, showed statistically significant inhibition of trypsin activity (p<0.001), and inhibition increased with higher concentrations of luteolin. As a result, we concluded that all analogs except for **9** do not act as non-specific inhibitors of trypsin activity (S10A Fig). In the second assay, well behaved inhibitors are expected to inhibit a target to the same degree even with increased target protein concentration. Potential aggregators are expected to show decreased inhibition with increased target protein concentration. No compounds showed significantly less PDE5 inhibition with an increase in PDE5 concentration (S10B Fig). Because compound **9** showed characteristics of a PAINS molecule in only one of the two assays, we cannot definitively conclude if compound **9** acts as a PAINS molecule. All other icariin analogs showed no characteristics of PAINS.

### Select icariin analogs exhibit enhanced inhibition potencies

**Fig 3:**
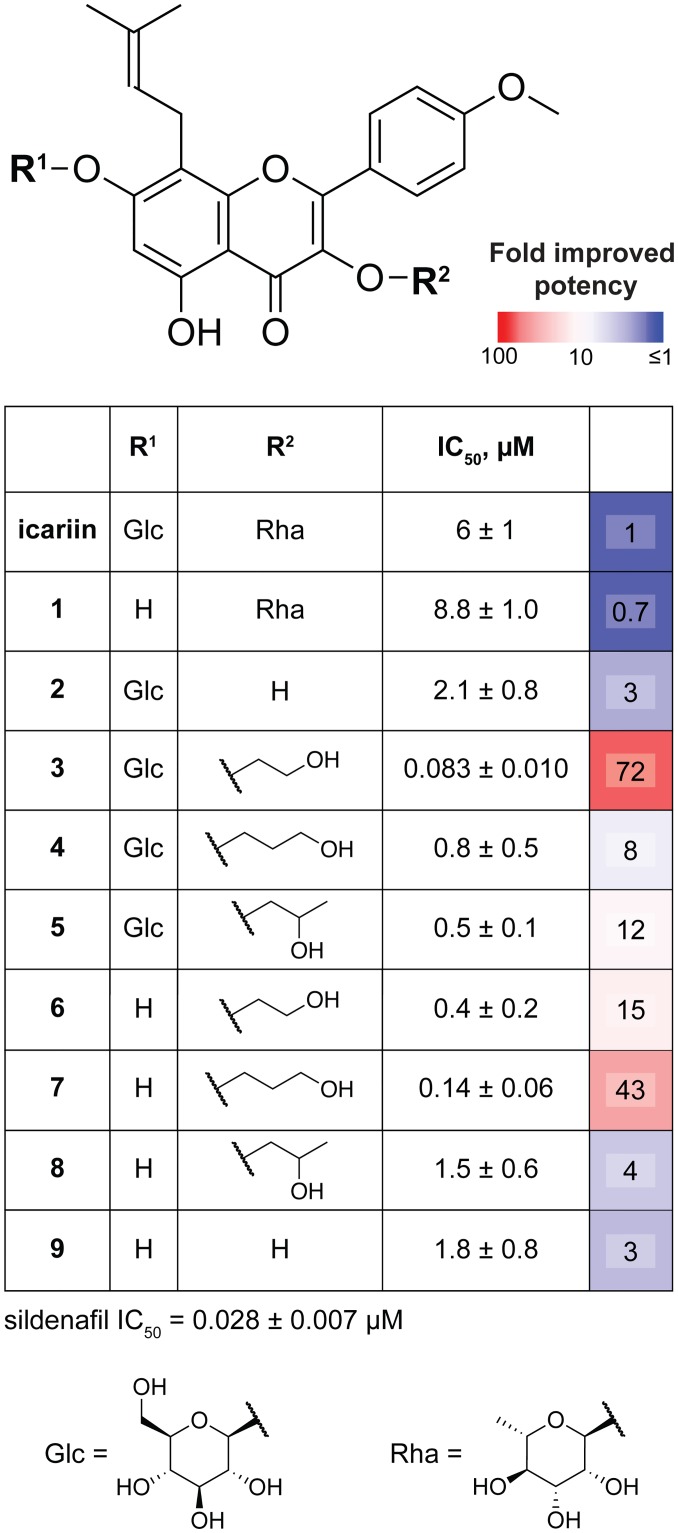
The 3-*O* and 7-*O* positions of icariin pharmacophore act synergistically to influence icariin analog PDE5 inhibition. IC_50_ values for icariin and icariin analogs **1–9** were obtained via *in vitro* inhibition assays using purified recombinant human PDE5. IC_50_ values represent mean (of at least three replicates) ± SEM. Fold improved potency refers to fold change in IC_50_ compared to icariin.

To evaluate their activity, we determined the IC_50_ of each of our nine analogs, icariin, and sildenafil using an *in vitro* PDE5 inhibition assay that measures GMP production from cGMP by purified recombinant human PDE5 (Fig 3). The IC_50_ values we obtained for sildenafil and icariin agree with previous reports (0.028 ± 0.007 μM and 6 ± 1 μM respectively) [17,18,20]. Replacing only the 7-*O*-glucose with a free hydroxyl (**1**) led to a slight loss in potency (IC_50_ 8.8 ± 1.0 μM) relative to icariin. This finding conflicts with the study by Dell’Agli et al. that reported an IC_50_ of 0.16 μM for **1** and a 34-fold improvement in PDE5 inhibition compared to icariin [18]. This discrepancy could be due to differences in assay design, such as the source of enzyme, since we used purified human PDE5 whereas Dell’Agli et al. used the lysate of COS-7 cells expressing human PDE5 in their enzyme activity assays. Replacing only the 3-*O*-rhamnose with a free hydroxyl (**2**) or replacing both sugars with a free hydroxyl (**9**) led to a 3-fold improvement in potency relative to icariin (IC_50_ 2.1 ± 0.8 and 1.8 ± 0.8 μM respectively). The IC_50_ value we obtained for **9** (icaritin) agrees with the value reported by Dell’agli et al [18]. These results support our initial hypothesis that removing the bulky and hydrophilic rhamnose leads to improved PDE5 inhibition and that retaining the glucose group does not negatively impact and may enhance PDE5 inhibition.

Compounds **6–8** allow us to evaluate the effects of alkylating the 3-OH in the absence of the 7-*O*-glucose on PDE5 inhibition. We observe that **8** (hydroxyisopropyl) is the least potent, **7** (hydroxypropyl) is the most potent, and **6** (hydroxyethyl) is in between, suggesting that compounds modified with linear alkanol groups are more potent inhibitors than those modified with branched alkanol groups. Compound **8** (IC_50_ 1.5 ± 0.6 μM) showed a 4-fold improvement over icariin, and **6** (IC_50_ 0.4 ± 0.2 μM) showed a 15-fold improvement. Compound **7** (IC_50_ 0.14 ± 0.06 μM) is our second most potent analog, showing a 42-fold improvement in inhibition. These low IC_50_ values, along with the large fold improvements in PDE5 inhibition for **6** and **7**, suggest that installation of a linear alkanol group, particularly a hydroxypropyl group, at the 3-OH is sufficient to markedly enhance PDE5 inhibition by an icariin analog.

Because compound **2** showed a 3-fold improvement in IC_50_ over icariin, we hypothesized that icariin analogs with both a 7-*O*-glucose and an alkanol at the 3-*O* position may lead to further improvement in potency. Compounds **3–5** allow us to evaluate this hypothesis. Combining the 7-*O*-glucose with a 3-*O*-alkanol led to an additional increase in PDE5 inhibition potency when the 3-OH is alkylated with either a hydroxyethyl group (**3**; IC_50_ 0.083 ± 0.010 μM) or a hydroxyisopropyl group (**5**; IC_50_ 0.5 ± 0.1 μM), representing a 5- and 3-fold increase in potency compared to the corresponding unglycosylated analogs **6** and **8**, respectively. This supports our hypothesis that combining the 7-*O*-glucose with a 3-*O*-alkanol further improves PDE5 inhibition. Compound **5** showed a 12-fold improvement in potency compared to icariin, whereas compound **3**, our most potent icariin analog with an IC_50_ of 0.083 ± 0.010 μM, showed a 72-fold increase in potency. Once again, the data suggest that a linear alkanol modification (**3**) is preferred over a branched alkanol modification (**5**). These observations stand in contrast to the results for **4** (IC_50_ 0.8 ± 0.5 μM). In this case, combining the 7-*O*-glucose with the 3-*O*-hydroxypropyl group led to a 6-fold decrease in potency compared to the corresponding unglycosylated compound **7** and only an 8-fold improvement in potency compared to icariin.

Evaluation of the correlations between IC_50_ values and analog structure reveal characteristics driving PDE5 inhibition by icariin analogs. The presence of a 7-*O*-glucose does not uniformly confer increased potency. Combining the 7-*O*-glucose with a 3-*O*-alkanol enhances (**3** and **5**) or reduces (**4**) the potency of our icariin analogs. These results suggest that the 3-*O* and 7-*O* functional groups act synergistically to affect icariin analog PDE5 inhibition potency. Even though the hydroxypropyl group in compound **4** is only one methylene group longer compared to **3** and **5**, it is possible that a slightly shorter functional group at the C3 position allows a better fit into the active site for icariin analogs with a 3-*O*-glucose. Since the PDE5 H-loop is reported to adopt alternative conformations when different inhibitors bind to PDE5, it is also possible that the shorter C3 groups combined with a 7-*O*-glucose (and in particular, the combination presented by compound **3**) induce a specific conformation within the PDE5 H-loop that promotes PDE5 binding and inhibition.

An additional interesting observation is that compound **7** has no 7-*O*-glucose group and only a 3-*O*-hydroxypropyl, but has an IC_50_ comparable to that of compound **3**, the most potent analog in this study. Furthermore, while combining the 7-*O*-glucose with a 3-*O*-alkanol did improve PDE5 inhibition for two icariin analogs (**3** and **5**), the unglycosylated icariin analogs with 3-*O*-alkanol groups (**6** and **7**) are already quite potent. These observations suggest that a hydrophobic group at the 3-*O* position may be the major characteristic driving inhibition. In summary, icariin analog PDE5 inhibition potency is driven by three features: 1) synergistic effects between the 3-*O* and 7-*O* positions, 2) hydrophobic groups at the 3-*O* position, and 3) a preference for linear alkanols at the 3-*O* position. These features are exemplified by compounds **3** and **7**, the two most potent analogs with IC_50_ values approaching the range of commercially available PDE5 inhibitors.

### Molecular modeling suggests features dictating icariin analog binding to PDE5

**Fig 4:**
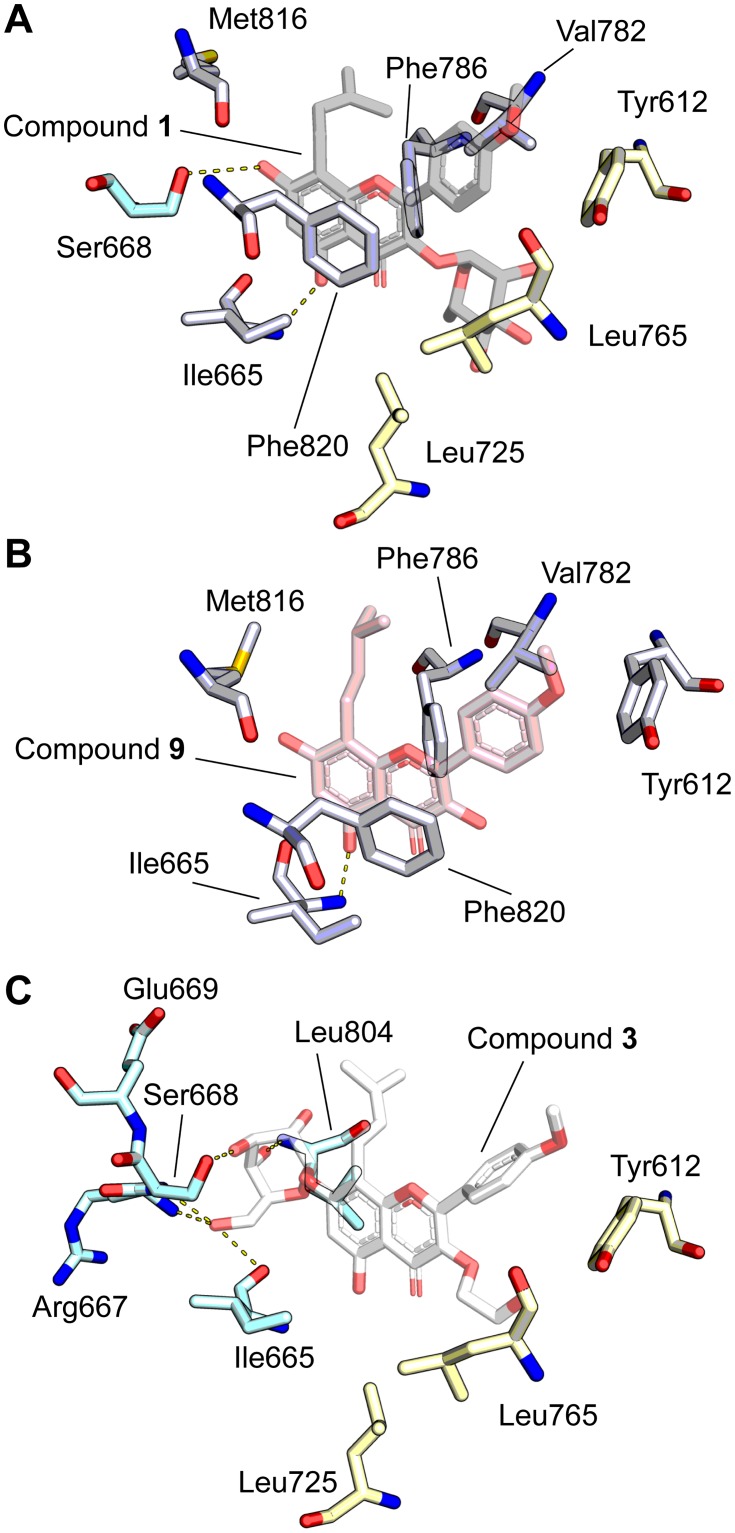
Molecular modeling of PDE5 with icariin analogs using PDB ID: 2H44 [20] as the starting model. (A) Crystal structure of compound **1** (grey) bound to PDE5 (PDB ID: 2H44) [20], highlighting ligand orientation and nearby interacting residues. Residues shared with panel B are white while residues shared with panel C are blue (interacts with 7-*O* position) and yellow (hydrophobic pocket; interacts with 3-*O* position). (B) Ligand docking model of compound **9** (pink) bound to PDE5 illustrates residues (white) that interact with conserved elements of the icariin-derived compounds, such as the flavone backbone and prenyl group. (C) Ligand docking model of compound **3** (white) bound to PDE5 illustrates residues that form hydrogen bonds with the 7-*O*-glucose (blue) and that form a hydrophobic pocket surrounding the 3-*O*-hydroxyethyl group (yellow). Hydrogen bonding is indicated with dashed yellow lines.

To rationalize the observed structure-activity relationships for our icariin analogs in the context of PDE5 inhibition, we used the existing co-crystal structure of PDE5 bound to **1** [20] (Fig 4A) as the starting model for ligand-docking simulations with our nine analogs. All icariin analogs share an identical core structure, and consequently, we found that there is a set of conserved interactions between the PDE5 residues and the various analogs (Fig 4B). For instance, a group of aromatic residues—Tyr612, Phe786, and Phe820—are involved in aromatic π−π interactions. Furthermore, the conserved prenyl group is bound in a hydrophobic pocket composed of Val782, Phe786, and Met816, and the conserved 5-OH is hydrogen bonding with the backbone amide of Ile665. These interactions are also observed in the existing crystal structure (Fig 4A, white residues) [20].

Next, we looked at the class of analogs containing the 7-*O*-glucose and found that a number of PDE5 residues interact with this functional group in our models (Fig 4C, blue residues). These residues, including Ile665, Arg667, Ser668, Glu669, and Leu804, are within hydrogen bonding distance (<3.5Å) (see S1 Table for list of residues that hydrogen-bond with each analog). Many of these residues are located in the H-loop, which is known to affect the ligand binding affinity based on its variable secondary structure and location [20–23]. There may be alternative or additional stabilizing interactions due to large conformational shifts in the H-loop, but they would not have been captured by our model. Obtaining crystal structures of PDE5 bound to **3**, **4**, or **5** would further clarify the 7-*O*-glucose contribution to PDE5 binding and inhibition.

Our IC_50_ data revealed that compounds with only a 3-*O*-alkanol (**6–8**) already show marked enhancement in PDE5 inhibition. This led us to explore the contribution of the other variable structural group, the 3-*O*-rhamnose in **1** and icariin and the 3-*O*-alkanol in **3–8**, to PDE5 binding. Both in the available crystal structure [20] and in our molecular modeling, we observe that a set of hydrophobic residues consisting of Tyr612, Leu725, and Leu765 surrounds the rhamnose binding site to create a hydrophobic pocket (Fig 4A and 4C, yellow residues). Although the rhamnose in **1** is within hydrogen-bonding distance to the surrounding residues His613 and Asp764 (S1 Table), this contribution may be nullified by the repulsive forces between the hydrophilic rhamnose and its hydrophobic binding area. In comparison, the 3-*O*-alkanol groups are hydrophobic, making them more suitable for placement in the hydrophobic pocket. Furthermore, the 3-*O*-alkanol in all analogs except for **4** and **9** are within hydrogen bonding distance to either His613 or Asp764 in our models. These observations explain the preference for analogs with a 3-*O*-alkanol (**3–8**) over those with a 3-*O*-rhamnose (icariin and **1**).

Asp764 is known to coordinate a zinc ion required for PDE5 catalytic activity and is predicted to play a role in cGMP hydrolysis [20,25,26]. In our models, we observed that the 3-*O*-hydroxypropyl on **4** loses the hydrogen bond to the potentially catalytically important Asp764 and instead forms a hydrogen bond with Leu725 while maintaining a similar number of hydrogen bonds to the 3-*O*-glucose as **3** and more hydrogen bonds overall than **7** (S1 Table). These observations may explain the unexpected finding that combining a glucose with the 3-*O*-hydroxypropyl in **4** decreased PDE5 inhibition potency. Additionally, the 3-*O*-alkanol group of **3**, **5**, **6**, or **7** is within hydrogen-bonding distance to Asp764. This result may explain why these four compounds are the most potent PDE5 inhibitors among our icariin analogs with a greater-than-10-fold improvement in inhibition compared to icariin.

Combined with the observation that compounds **6** and **7** have low IC_50_ values, these modeling results support the greater contribution of 3-*O*-alkanols over that of the 7-*O*-glucose with regard to binding affinity. Therefore, the lower potency of compound **9** can be explained by its lack of functional groups at both the 3-*O* and 7-*O* positions, resulting in a loss of stabilizing interactions with PDE5. We note that our results do not discern why there is a preference for a linear alkanol modification over a branched alkanol at the 3-OH position. There might be additional conformational changes not captured in our models that account for these observations.

### Viability of selected human cell lines is minimally affected by treatment with compounds 3 and 7

To test if a selection of our icariin analogs affects cellular viability and health, we treated four human cell lines that express PDE5 protein (S11A and S12 Figs) with 0.01–10 μM icariin, compounds **2–7**, or sildenafil, and measured mitochondrial viability with resazurin. The four cell lines tested were the neuroblastoma cell line SH-SY5Y (SY5Y, ATCC CRL-2266), the melanoma cell line SK-MEL-5 (SKMEL-5, ATCC HTB-70), the immortalized fibroblast cell line BJ-hTERT [27], and the small cell lung cancer cell line NCI-H187 (ATCC CRL-5804). Treatment with icariin, compounds **3**, **5**, **7**, or sildenafil resulted in minimal (viability >70%) to no effect on cell viability even at the highest concentration tested (10 μM). In contrast, we observed a marked reduction in viability (viability of 70% or lower) in at least one cell line treated with compounds **2**, **4**, or **6** (S11B–S11E Fig). In particular, compound **6** treatment led to this marked reduction in viability of all four cell lines tested. One possible explanation for our results is that these compounds modulate additional molecular targets besides PDE5. However, we note that cGMP is known to regulate the expression of genes that control cell growth, proliferation and apoptosis [28], making it unsurprising that we observe effects on viability as a result of PDE5 inhibition. While additional assays are needed to fully elucidate the impact of icariin analog treatment on cellular growth and health, compounds **3** and **7**, our two most potent analogs, exhibit minimal effects on mitochondrial viability in the tested human cell lines.

### Enzyme kinetics reveals *K*_*i*_ and mode of inhibition for compounds 3 and 7

**Fig 5:**
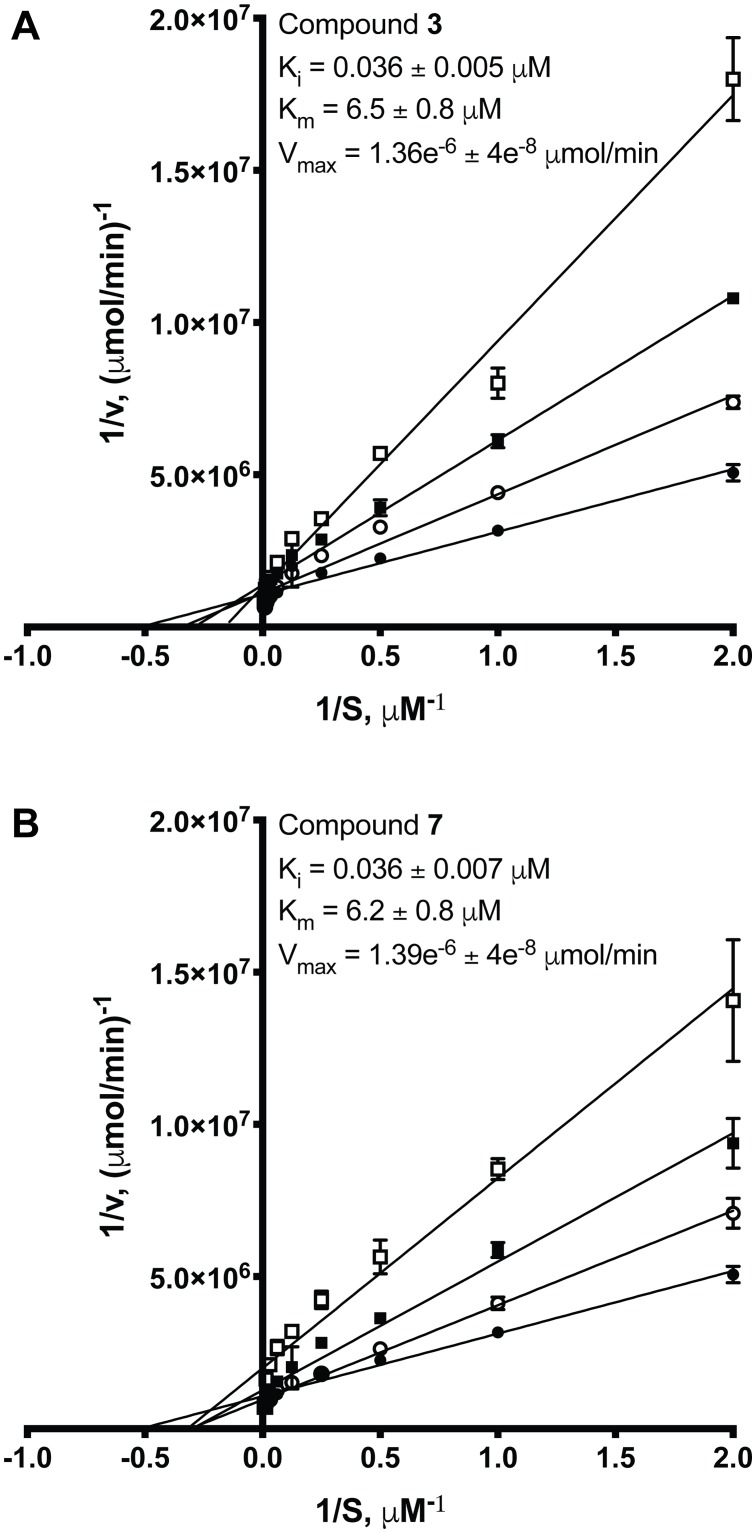
Lineweaver-Burk plots from *in vitro* PDE5 kinetics assays with compound 3 (A) or 7 (B). (A) Compound **3** acts as a competitive inhibitor with an inhibition constant (*K*_*i*_) in the mid-nanomolar range. (B) Compound **7** inhibition may be more complicated. The *K*_*i*_ for a competitive inhibition model is also in the mid-nanomolar range. ● is no inhibitor, ○ is 0.015 μM inhibitor, ■ is 0.05 μM inhibitor, and □ is 0.2 μM inhibitor. Each data point and *K*_*i*_, *K*_*m*_, and *V*_*max*_ values represent means (from at least three replicates) ± SEM. *K*_*i*_, *K*_*m*_, and *V*_*max*_ values were determined by nonlinear regression analysis.

The crystal structure of PDE5 in complex with **1** suggests that **1** binds to the active site as a competitive inhibitor [20]. However, to the best of our knowledge, the inhibition constant (*K*_*i*_) or the mode of inhibition in the context of PDE5 inhibition for any icariin analog has not been determined. To further characterize our two most potent analogs, we determined the *K*_*i*_ and the inhibition mode of **3** and **7** using an *in vitro* PDE5 kinetics assay. Compound **3** acts as a competitive inhibitor, as demonstrated by the Lineweaver-Burk plot (Fig 5A) and by comparing the fits of different inhibition models with Akaike’s Information Criteria (AICc) analysis (>99% probability that the competitive inhibition model is correct compared to a noncompetitive or uncompetitive model). For compound **7**, AICc analysis identifies competitive inhibition as the best fitting model for the kinetics data (>99% probability that the competitive inhibition model is correct compared to a noncompetitive or uncompetitive model). The Lineweaver-Burk plot (Fig 5B) hints that the mode of inhibition may not be purely competitive, since the plot does not entirely conform to the characteristics of a competitive inhibitor. Both noncompetitive and mixed model inhibition do not fit compound **7** kinetics data better than the competitive inhibition model as determined by AICc analysis or visual inspection of the Michaelis-Menten plots. The *K*_*i*_ values determined by nonlinear regression analysis using the competitive inhibition model for **3** and **7** are in the mid-nanomolar range: 0.036 ± 0.005 μM and 0.036 ± 0.007 μM, respectively. The *K*_*m*_ determined for PDE5 is 6.3–6.5 ± 0.8 μM, which agrees with previously published reports [6].

### Compounds 3 and 7 decrease GMP levels in the human BJ-hTERT cell line

**Fig 6:**
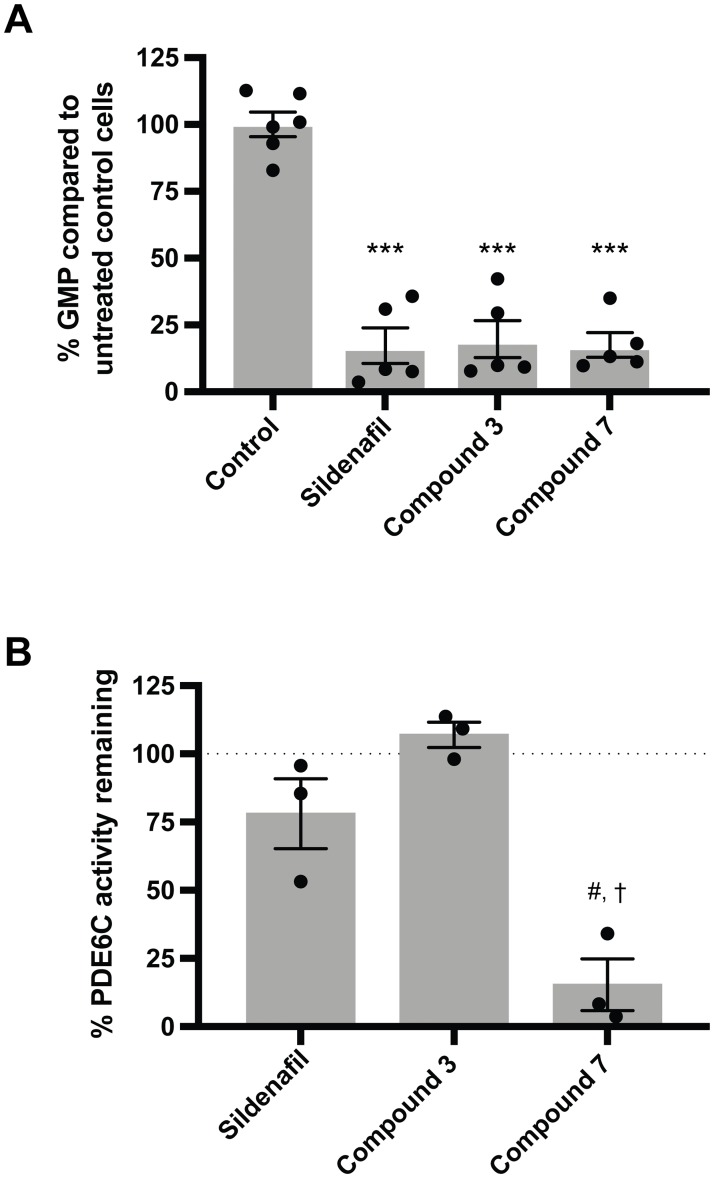
Effect of compound 3 and 7 treatment on intracellular GMP levels and on PDE6C activity. (A) Relative GMP levels in the human cell line BJ-hTERT after treatment with 20 μM sildenafil, **3**, or **7**. Circles are values from individual samples, grey bars are means, and error bars are SEM. Three independent assays were conducted. ***p<0.001 *vs* control (one-way ANOVA with Tukey’s multiple comparisons test). Range of average GMP concentration in control samples (no drug treatment, 4E5 cells): 0.1 ± 0.001 to 3 ± 0.2 μM. (B) Recombinant human PDE6C activity (measured as GMP production) when treated with sildenafil, **3**, or **7** at 50x the PDE5 IC_50_ concentration. % PDE6C activity remaining was calculated as described in the materials and methods section. Circles are values from three replicates, grey bars are mean, and error bars areSEM. #p<0.01 *vs* hypothetical mean of 100%; sildenafil and compound **3** were not significant (one sample *t* test). †p<0.05 *vs* sildenafil (p = 0.02) and *vs* compound **3** (p<0.001); sildenafil *vs* compound **3** was not significant (two-sample *t* test). Average untreated control reaction velocity: 1E6 ± 0.2E6 μmol min^-1^ U^-1^.

To determine if compounds **3** and **7** can have an effect on intracellular PDE5 activity, we measured the GMP concentration in the human cell line BJ-hTERT after treatment with sildenafil, **3**, and **7** for 1 hour. BJ-hTERT is an immortalized non-transformed human fibroblast cell line from fetal foreskin with high PDE5 expression (S11A and S12 Figs) [27]. PDE5 inhibitors are expected to decrease GMP concentration in cells. After treatment with 20 μM of inhibitor for 1 hour, cellular GMP concentrations were significantly reduced to 7 ± 1, 9 ± 0.6, and 13 ± 2% of the GMP concentration in untreated control cells for sildenafil, **3**, and **7**, respectively (p<0.001, Fig 6A). Cells appeared healthy upon visual inspection under a light microscope after drug treatment. This result suggests that compounds **3** and **7** can inhibit intracellular PDE5 and decrease cellular GMP levels in cultured human cells.

### Compound 3 exhibits specificity for PDE5 over PDE6C

A major side effect associated with the use of commercial PDE5 inhibitors is visual impairment. This results from off-target inhibition of PDE6, cGMP phosphodiesterase isoforms expressed in the rods (PDE6A, PDE6B) and cones (PDE6C) of the retina that regulate vision signaling [8,9]. To test for specificity, we assayed for inhibition of partially purified recombinant human PDE6C by sildenafil, **3**, and **7** at 50× their PDE5 IC_50_ (Fig 6B). At this concentration, **3** and sildenafil still do not show any statistically significant PDE6C inhibition. Furthermore, while not statistically significant, **3** appears to show less PDE6C inhibition compared to sildenafil (78 ± 13% and 107 ± 5% PDE6C activity remaining for sildenafil and **3**, respectively). However, at 50× the IC_50_ concentration, **7** significantly inhibits PDE6C activity (15 ± 9% PDE6C activity remaining; p<0.01 *vs* the theoretical uninhibited mean of 100%, p = 0.02 *vs* sildenafil, p<0.001 *vs* **3**). This result suggests that while a hydrophobic group at the 7-*O* position readily confers PDE5 inhibition potency, the presence of another functional group (such as the 3-*O*-glucose in **3**) is needed to confer specificity.

## Conclusions

Using a series of naturally occurring and novel semi-synthetic icariin analogs, we explored the structure-activity relationships between PDE5 inhibition and the icariin pharmacophore. *In vitro* inhibition assays revealed that while a flexible, linear, hydrophobic group at the 7-*O* position is sufficient to increase PDE5 inhibition potency by up to 43-fold compared to icariin, the 3-*O* and 7-*O* positions can act synergistically to improve the potency of PDE5 inhibition up to 72-fold compared to icariin. Molecular modeling provided additional support for the structure-activity relationships revealed by IC_50_ measurements, wherein the 7-*O*-glucose may participate in hydrogen bonding with PDE5 active-site-entrance residues, and the 3-*O*-alkanols confer favorable interactions with a hydrophobic pocket of the active site. Compounds **3** and **7** are our most potent analogs with IC_50_ and *K*_*i*_ values in the mid-nanomolar range, approaching those of the synthetic PDE5 inhibitors. *In vitro* kinetics assays suggest that **3** inhibits PDE5 competitively and **7** inhibits PDE5 by a partially competitive model. Both **3** and **7** can reduce GMP levels in the human BJ-hTERT cell line similarly to sildenafil, demonstrating their effectiveness at inhibiting the activity of intracellular PDE5. While compound **3** shows specificity for PDE5 over PDE6C, compound **7** does not show this specificity, suggesting the need for functional groups at both the 3-*O* and 7-*O* positions to confer specificity. Both **3** and **7** show minimal effects on the viability of four human cell lines, and the synthesis of compounds **3** and **7** had high yields, establishing them as promising leads as new PDE5 inhibitors poised for further preclinical and clinical development.

## Materials and methods

### General procedures

All chemicals were purchased from Sigma-Aldrich, unless otherwise specified. Purified recombinant human PDE5 (catalytic domain, amino acids 537–875) was purchased from Abcam. Partially purified recombinant human PDE6C (full-length protein) was purchased from SB Drug Discovery. Solution NMR spectra were recorded on a Bruker AVANCE-400 NMR spectrometer with a Spectro Spin superconducting magnet in the Massachusetts Institute of Technology, Department of Chemistry Instrumentation Facility (MIT DCIF). HPLC-UV-MS analysis was done on an Ultimate 3000 liquid chromatography system (Dionex), equipped with a 150 mm C18 Column (Kinetex 2.6 μm silica core shell C18 100Å pore, Phenomenex) and coupled to an UltiMate 3000 diode-array detector (DAD) in-line UV-Vis spectrophotometer (Dionex) and a TSQ Quantum Access MAX triple-quadrupole mass spectrometer (Thermo-Scientific). Solvents for liquid chromatography mass spectrometry were Optima^®^ LC-MS grade (Fisher Scientific) or LiChrosolv^®^ LC-MS grade (Millipore). The column oven and autosampler tray were held at 30 °C and 10 °C respectively. The mass spectrometer was operated in positive mode with the spray voltage set to 4.0 kV, the heated capillary held at 380 °C, and the HESI probe held at 400 °C. The sheath gas flow was set to 60 units, the auxiliary gas flow was set to 30 units, and the sweep gas flow was set to 0 unit. The mobile phases were solvent A (water, 0.1% formic acid) and solvent B (acetonitrile, 0.1% formic acid). Unless otherwise specified, the chromatographic gradient was run at a flow rate of 0.7 mL/min as follows: 0–1 minutes 5% B, 1–15 minutes 5–80% B, 15–25 minutes 95% B, 25–30 minutes 5% B. The purity of synthesized compounds were analyzed by HPLC-UV-MS in full scan mode. All synthesized compounds exhibited purities over 95%.

The synthesis of each analog is described below. Icariin used in synthesis was purchased from TCI Chemicals.

### Synthesis of icariin derivatives 1–9

Icariside II (**1**). Synthesis was carried out according to a modified method of Dell’Agli et al [18]. A solution of icariin (100 mg) in DMSO (1 mL) was added to 0.1 M Na acetate buffer (pH 5.7, 50 mL) at 37 °C containing cellulase (0.6 U per mg icariin). The suspension obtained was stirred at 37 °C overnight. Then the mixture was extracted with EtOAc (3 × 200 mL). The organic layer was dried over anhydrous Na_2_SO_4_ and evaporated under reduced pressure. The residue was purified by column chromatography on LH20 (MeOH) to afford **1** (60 mg, 84% yield). ^1^H NMR (DMSO-*d*_*6*_, 400 MHz), *δ* 12.52 (1H, s, OH-5), 7.84 (2H, d, *J* = 8.8 Hz, H-2’, 6’), 7.11 (2H, d, *J* = 8.8 Hz, H-3’, 5’), 6.31 (1H, s, H-6), 5.15 (1H, t, *J* = 6.4 Hz, H-12), 4.63–5.26 (3H, m, Rha-OH), 3.05–4.64 (5H, m, Rha-H), 3.85 (3H, s, OCH_3_), 3.43 (2H, d, *J* = 6.8 Hz, H-11), 1.70, 1.62 (3H, 3H, s, CH_3_-14, 15), 0.77 (3H, d, *J* = 6.0 Hz, Rha-CH_3_). ^13^C NMR (DMSO-*d*_*6*_, 100 MHz), *δ* 178.4, 162.2, 161.7, 159.3, 157.2, 154.2, 134.9, 131.5, 130.9, 122.8, 122.7, 114.5, 106.4, 104.6, 102.4, 98.8, 71.6, 71.1, 70.8, 70.5, 56.0, 25.9, 21.6, 18.3, 17.9 ppm. ESIMS (positive-ion mode) m/z 515 [M + H]^+^. HPLC purity: 97.23%, retention time = 10.48 min.

Icariside I (**2**). Synthesis was carried out according to a method of Shen [29]. A solution of icariin (800 mg) in ethanol (11 mL) was added to 11 mL 5% aqueous sulfuric acid solution, stirred for 24 hours at 50 °C, cooled to room temperature, and concentrated under reduced pressure to remove ethanol. Then the mixture was extracted with EtOAc (3 × 200 mL) and washed with saturated brine and NaHCO_3_ solution (2 × 200 mL). The organic layer was dried over anhydrous Na_2_SO_4_ and evaporated under reduced pressure. The residue was purified by column chromatography on LH20 (MeOH) to afford **2** (450 mg, 60% yield). ^1^H NMR (DMSO-*d*_*6*_, 400 MHz), *δ* 12.43 (1H, s, OH-5), 9.61 (1H, s, OH-3), 8.14 (2H, d, *J* = 8.8 Hz, H-2’, 6’), 7.13 (2H, d, *J* = 8.8 Hz, H-3’, 5’), 6.60 (1H, s, H-6), 5.21 (1H, t, *J* = 6.8 Hz, H-12), 4.63–5.34 (4H, m, Glu-OH), 5.00 (1H, d, *J* = 6.8 Hz, Glu-H), 3.14–3.76 (6H, m, Glu-H), 3.85 (3H, s, OCH_3_), 3.43 (2H, d, *J* = 6.8 Hz, H-11), 1.77, 1.62 (3H, 3H, s, CH_3_-14, 15). ^13^C NMR (DMSO-*d*_*6*_, 100 MHz), *δ* 177.0, 161.1, 160.6, 159.0, 153.2, 147.4, 136.7, 131.6, 129.8, 123.9, 122.8, 114.6, 108.5, 105.0, 100.9, 97.9, 77.6, 77.1, 73.8, 70.1, 61.1, 55.9, 26.0, 21.9, 18.4 ppm. ESIMS (positive-ion mode) m/z 531 [M + H]^+^. HPLC purity: 96.59%, retention time = 9.92 min.

#### General procedure A for the synthesis of compounds 3–5

Synthesis of compounds **3–5** was carried out according to a modified method of Dell’Agli [18]. A stirred suspension of **2**, a bromylated alkanol, and anhydrous K_2_CO_3_ in dry acetone (35 mL) was refluxed for 8 h. After the reaction mixture cooled down, the solvent was evaporated under reduced pressure, the residue suspended in 200 mL H_2_O, and extracted with EtOAc (3 × 200 mL). The organic layer was dried over anhydrous Na_2_SO_4_, evaporated under reduced pressure. The residue was purified by column chromatography on LH20 (MeOH) to afford the icariin analog.

3-*O*-(2-Hydroxyethyl)-Icariside I (**3**). Synthesis followed general procedure A starting with **2** (1 equiv), 2-bromoethanol (22.4 equiv), and anhydrous K_2_CO_3_ (11.5 equiv) to afford **3** (40% yield). ^1^H NMR (DMSO-*d*_*6*_, 400 MHz), *δ* 12.63 (1H, s, OH-5), 8.17 (2H, d, *J* = 8.8 Hz, H-2’, 6’), 7.12 (2H, d, *J* = 8.8 Hz, H-3’, 5’), 6.62 (1H, s, H-6), 5.19 (1H, t, *J* = 7.2 Hz, H-12), 4.82 (1H, t, *J* = 4.8 Hz, OH-17), 4.62–5.34 (4H, m, Glu-OH), 5.00 (1H, d, *J* = 6.0 Hz, Glu-H), 3.17–3.74 (6H, m, Glu-H), 4.07 (2H, t, *J* = 4.8 Hz, H-17), 3.66 (2H, t, *J* = 5.2 Hz, H-16), 3.87 (3H, s, OCH_3_), 3.43 (2H, m, H-11), 1.75, 1.62 (3H, 3H, s, CH_3_-14, 15). ^13^C NMR (DMSO-*d*_*6*_, 100 MHz), *δ* 179.1, 161.9, 160.9, 159.5, 156.1, 153.4, 137.6, 131.7, 130.8, 122.9, 122.7, 114.6, 108.7, 106.1, 101.0, 98.4, 77.7, 77.1, 74.3, 73.8, 70.1, 61.1, 60.7, 56.0, 26.0, 21.9, 18.4 ppm. ESIMS (positive-ion mode) m/z 575 [M + H]^+^. HPLC purity: 95.02%, retention time = 8.99 min.

3-*O*-(3-Hydroxypropyl)-Icariside I (**4**). Synthesis followed general procedure A starting with **2** (1 equiv), 3-bromo-1-propanol (5.9 equiv), and anhydrous K_2_CO_3_ (5.9 equiv) to afford **4** (48% yield). ^1^H NMR (DMSO-*d*_*6*_, 400 MHz), *δ* 12.64 (1H, s, OH-5), 8.05 (2H, d, *J* = 9.2 Hz, H-2’, 6’), 7.15 (2H, d, *J* = 9.2 Hz, H-3’, 5’), 6.62 (1H, s, H-6), 5.19 (1H, t, *J* = 6.8 Hz, H-12), 4.46–5.34 (4H, m, Glu-OH), 4.99 (1H, d, *J* = 6.8 Hz, Glu-H), 3.17–3.74 (6H, m, Glu-H), 4.62 (1H, t, *J* = 6.0 Hz, OH-18), 4.06 (2H, t, *J* = 6.8 Hz, H-18), 3.49 (2H, t, *J* = 6.0 Hz, H-16), 3.87 (3H, s, OCH_3_), 3.43 (2H, m, H-11), 1.79 (2H, t, *J* = 6.8 Hz, H-17), 1.73, 1.62 (3H, 3H, s, CH_3_-14, 15). ^13^C NMR (DMSO-*d*_*6*_, 100 MHz), *δ* 177.0, 161.1, 160.6, 159.1, 153.2, 136.7, 131.6, 130.6, 129.8, 122.8, 122.7, 114.6, 108.5, 105.0, 100.9, 97.6, 77.6, 77.1, 73.8, 70.1, 61.1, 58.0, 55.9, 55.8, 26.0, 21.9, 18.4 ppm. Some carbon signals (e.g., C-10) were not observed due to the low amount of sample available. ESIMS (positive-ion mode) m/z 589 [M + H]^+^. HPLC purity: 95.45%, retention time = 9.65 min.

3-*O*-(2-Hydroxypropyl)-Icariside I (**5**). Synthesis followed general procedure A starting with **2** (1 equiv), 1-bromo-2-propanol (7.8 equiv), and anhydrous K_2_CO_3_ (5.9 equiv) to afford **5** (10% yield). ^1^H NMR (DMSO-*d*_*6*_, 400 MHz), *δ* 8.13 (2H, d, *J* = 8.8 Hz, H-2’, 6’), 7.13 (2H, d, *J* = 8.8 Hz, H-3’, 5’), 6.61 (1H, s, H-6), 5.19 (1H, t, *J* = 6.4 Hz, H-12), 4.41–5.35 (4H, m, Glu-OH), 5.00 (1H, d, *J* = 6.8 Hz, Glu-H), 3.17–3.73 (6H, m, Glu-H), 4.84 (1H, m, H-17), 3.86 (2H, m, H-16), 3.86 (3H, s, OCH_3_), 3.43 (2H, m, H-11), 1.74, 1.62 (3H, 3H, s, CH_3_-14, 15). 1.08 (1H, d, *J* = 6.4 Hz, H-18). ^13^C NMR data were not obtained due to the low amount of compound **5** available. ESIMS (positive-ion mode) m/z 589 [M + H]^+^. HPLC purity: 97.22%, retention time = 9.56 min.

#### General procedure B for the preparation of compounds 6–9

Synthesis of compounds **6–9** was similar to the preparation of **1** but modified. A solution of compound **3, 4**, or **5** (5 mg) or **2** (10 mg) in DMSO (1 mL) was added to 0.1 M Na acetate buffer (pH 5.7, 50 mL) at 37 °C containing cellulase (2 U per mg **3**, **4**, or **5**; 1 U per mg **2**). The suspension obtained was stirred at 37 °C overnight. Then the mixture was extracted with EtOAc (3 × 10 mL). The organic layer was dried over anhydrous Na_2_SO_4_ and evaporated under reduced pressure. The residue was purified by column chromatography on LH20 (MeOH) to afford the icariin analog.

3-*O*-(2-Hydroxyethyl)-Icaritin (**6**). Synthesis followed general procedure B starting with a solution of **3** to afford **6** (1.8 mg, 56% yield). ^1^H NMR (DMSO-*d*_*6*_, 400 MHz), *δ* 12.56 (1H, s, OH-5), 8.13 (2H, d, *J* = 8.8 Hz, H-2’, 6’), 7.10 (2H, d, *J* = 9.2 Hz, H-3’, 5’), 6.23 (1H, s, H-6), 5.17 (1H, t, *J* = 6.8 Hz, H-12), 4.03 (2H, t, *J* = 4.8 Hz, H-16), 3.64 (2H, t, *J* = 4.8 Hz, H-17), 3.85 (3H, s, OCH_3_), 3.43 (2H, m, H-11), 1.73, 1.62 (3H, 3H, s, CH_3_-14, 15). ^13^C NMR data were not obtained due to the low amount of compound **6** available. ESIMS (positive-ion mode) m/z 413 [M + H]^+^. HPLC purity 95.08%, retention time = 12.27 min.

3-*O*-(3-Hydroxypropyl)-Icaritin (**7**). Synthesis followed general procedure B starting with a solution of **4** to afford **7** (1.0 mg, 50% yield). ^1^H NMR (DMSO-*d*_*6*_, 400 MHz), *δ* 12.57 (1H, s, OH-5), 8.01 (2H, d, *J* = 8.8 Hz, H-2’, 6’), 7.12 (2H, d, *J* = 8.8 Hz, H-3’, 5’), 6.13 (1H, s, H-6), 5.17 (1H, t, *J* = 6.4 Hz, H-12), 4.02 (2H, t, *J* = 6.8 Hz, H-18), 3.47 (2H, t, *J* = 6.4 Hz, H-16), 3.85 (3H, s, OCH_3_), 3.34 (2H, d, *J* = 6.4 Hz, H-11), 1.77 (2H, t, *J* = 6.4 Hz, H-17), 1.72, 1.62 (3H, 3H, s, CH_3_-14, 15). ^13^C NMR data were not obtained due to the low amount of compound **7** available. ESIMS (positive-ion mode) m/z 427 [M + H]^+^. HPLC purity 99.62%, retention time = 12.71 min.

3-*O*-(2-Hydroxypropyl)-Icaritin (**8**). Synthesis followed general procedure B starting with a solution of **5** to afford **8** (2.4 mg, 62% yield). ^1^H NMR (DMSO-*d*_*6*_, 400 MHz), *δ* 12.49 (1H, s, OH-5), 8.04 (2H, d, *J* = 8.8 Hz, H-2’, 6’), 7.08 (2H, d, *J* = 8.8 Hz, H-3’, 5’), 5.76 (1H, s, H-6), 5.15 (1H, m, H-12), 3.74 (1H, m, H-17), 3.87 (2H, m, H-17), 3.84 (3H, s, OCH_3_), 3.28 (2H, m, H-11), 1.71, 1.61 (3H, 3H, s, CH_3_-14, 15). 1.04 (1H, d, *J* = 6.4 Hz, H-18). ^13^C NMR data were not obtained due to the low amount of compound **8** available. ESIMS (positive-ion mode) m/z 427 [M + H]^+^. HPLC purity 98.26%, retention time = 13.11 min.

Icaritin (**9**). Synthesis followed general procedure B starting with a solution of **2** to afford **9** (6.7 mg, 68% yield). ^1^H NMR (DMSO-*d*_*6*_, 400 MHz), *δ* 12.36 (1H, s, OH-5), 8.12 (2H, d, *J* = 8.8 Hz, H-2’, 6’), 7.12 (2H, d, *J* = 8.8 Hz, H-3’, 5’), 6.29 (1H, s, H-6), 5.18 (1H, t, *J* = 6.8 Hz, H-12), 3.85 (3H, s, OCH_3_), 3.43 (2H, d, *J* = 6.8 Hz, H-11), 1.75, 1.63 (3H, 3H, s, CH_3_-14, 15). ^13^C NMR data were not obtained due to the low amount of compound **9** available. ESIMS (positive-ion mode) m/z 369 [M + H]^+^. HPLC purity 97.88%, retention time = 13.68 min.

### Enzyme inhibition and kinetics measurement

STRENDA guidelines [30] were considered in the description of the following enzyme activity assays. PDE5 inhibition and kinetics assays were carried out according to the method of Kincaid and Manganiello [31] and Dell’Agli et al [32]. 1 nM recombinant human PDE5 (catalytic domain consisting of amino acids 537–875, Abcam, ab125581) was pre-incubated with PDE5 inhibitors (synthesized in-house as described above or purchased from Selleck Chemicals) in 40 mM tris-hydrochloric acid, pH 7.8, containing 10 mM MgCl_2_. For inhibition assays, inhibitor concentrations used ranged from 0.001–150 μM. For kinetics assays, 0.015, 0.05, and 0.2 μM of compounds **3** and **7** were used. PDE5 inhibitors were added to each reaction using a Hewlett-Packard D300 Digital Dispenser (Tecan). Maximum DMSO concentration was 0.1% of the final reaction volume for all samples. After incubation at 25 °C for 5 minutes to allow interaction between PDE5 and drugs, the reaction was started with the addition of 0.3 μM cGMP (Enzo Life Sciences, Inc.) and incubated at 25 °C for 15 minutes. The final reaction volume was 100 μL. Reactions were stopped with the addition of 100 μL of 0.1 M HCl and neutralized by adding 0.1 M NaOH until the pH was 7. Under these reaction conditions, 10–15% of cGMP was consumed in no-drug control reactions, and samples taken at different time points between 0–15 minutes showed that the reaction remained linear. The amount of GMP produced was measured by HPLC-UV-MS as described in the general procedures. Reaction mixture components were separated with a ramp gradient of solvent A and B run at a flow rate of 0.5 mL/min as follows: 0–4 minutes 0% B, 4–7 minutes 0–95% B, 7–8 minutes 95% B, 8–11.1 minutes 0% B. GMP was measured using selected reaction monitoring in positive mode for a centroid center mass of 152.

A calibration curve using known concentrations of GMP was generated using the HPLC-UV-MS method described above to quantify the absolute amount of GMP in each reaction (S13 Fig). The linear range was from 5.9 nM to 3 μM. The limit of detection (LOD), defined as the lowest concentration producing a detectable peak above background, was 0.1 nM. The lower limit of quantification (LLOQ), defined as the lowest concentration used to generate a standard curve with r^2^ > 0.95, was 5.9 nM. The upper limit of quantification (ULOQ), defined as the highest concentration used to generate a standard curve with r^2^ > 0.95, was 3 μM. A GMP calibration curve was generated with each individual run of the HPLC-UV-MS.

To measure the IC_50_ as a result of treatment with each icariin analog, the *in vitro* inhibition enzyme assay as described above was performed, and GMP levels were normalized to no-drug controls and multiplied by 100 to obtain the percent PDE5 activity. IC_50_ mean ± SEM values were determined by nonlinear-regression fitting of the percent PDE5 activity data using the [inhibitor] *vs* response–variable slope (four parameters) equation in Prism (version 7.0a and version 8). Top value was constrained to 100, all other parameters were unconstrained. To obtain the dose-response curves for visual confirmation of the best-fit line, we executed a nonlinear regression fitting using the log(inhibitor) vs. response—variable slope (four parameters) equation in Prism. *K*_*i*_, *K*_*m*_, and *V*_*max*_ values were determined by nonlinear regression fitting (least squares fit) of kinetics data using the competitive inhibition equation in Prism. AICc analysis (built into Prism) was used to confirm the best-fitting inhibition model. For both compounds **3** and **7**, the AICc analysis calculated a >99% probability that the competitive inhibition model fits best (compared to either noncompetitive, uncompetitive, or mixed models of inhibition). Inhibition models were also compared via visual inspection of the fit of the calculated curves on the Michaelis-Menten plots. Keeping cGMP concentration at less than 1/10 of the *K*_*m*_ for PDE5 and staying within a linear reaction range ensure that the reaction remains within Michaelis-Menten model assumptions and that the IC_50_ is approximately equivalent to the inhibition constant or *K*_*i*_ (as substrate concentration decreases, IC_50_ value approaches *K*_*i*_ value) [33].

PDE6C inhibition was measured using the same method as described for PDE5 with modifications. 0.25 U of partially purified recombinant full-length human PDE6C (partially purified from insect cells, SB Drug Discovery) was pre-incubated with sildenafil (1.5 or 7.5 μM), compound **3** (5 μM), or compound **7** (5 μM) at 25 °C for 15 minutes. The concentration of each inhibitor was approximately 50x (and 250x for sildenafil) the PDE5 IC_50_. The final reaction volume was 50 μL. Maximum DMSO concentration was 0.25% of the total reaction volume. Reactions were started by adding 14 μM cGMP and incubated at 25 °C for 1 hour. Reactions were stopped as described above for PDE5 enzyme assays, and GMP was quantified as described above. Because PDE6C was partially purified and the enzyme mixture contained other proteins, there was GMP production from non-PDE6C sources. To account for this, samples treated with the highest concentration of sildenafil was assumed to represent 100% PDE6C inhibition, and any GMP production was assumed to result from non-PDE6C activity. The amount of GMP produced in these samples was subtracted from all samples before normalizing against untreated controls.

### PAINS assays

Icariin and compounds **1–9** were assayed against bovine trypsin (Sigma-Aldrich, T1426) at 1x and 10x the IC_50_ concentration. Compounds **3** and **7** were also assayed at 50x and 250x the IC_50_ concentration. In assay buffer (50 mM Tris-HCl, 20 mM CaCl_2_, pH 8.19 with or without 0.5% igepal), 0.002 mg freshly made trypsin is incubated with drugs for 5 minutes at 25 °C. 1 mM of substrate (Nα-benzoyl-L-arginine 4-nitroanilide hydrochloride, Sigma-Aldrich, B3133) is added to start the reaction. Final reaction volume is 100 μl. Product formation is monitored by increasing absorbance at 410 nm at 25 °C for 30 minutes in kinetic mode using a SpectraMax M5 plate reader (Molecular Devices). All values were measured in duplicate.

To assay the effect of increasing PDE5 concentration on icariin and icariin analogs, the PDE5 inhibition assay described in the methods section was done with modifications. Compounds at approximately 1x the PDE5 IC_50_ concentration were incubated with 1 or 2 nM PDE5, and GMP production was measured as described. All values were measured in triplicate.

### Ligand docking

The crystal structure of PDE5A1 in complex with icariside II (PDB ID: 2H44), removed of all non-protein atoms, was used as the starting model for ligand docking. Structures of icariin derivatives were generated by modifying the icariside II model from the crystal structure (PDB ligand: 7CA) in Avogadro: an open-source molecular builder and visualization tool, Version 1.2.0 (www.avogadro.cc) or MarvinSketch Version 17.18.0, ChemAxon (www.chemaxon.com) [34]. To constrain search space to the PDE5 binding site, Coot Version 0.8.8 was used to align icariin analog structures with icariside II in the 2H44 PDB structure and save the new ligand coordinates [35,36]. The protein and ligand were then input into ROSIE,[37] the Rosetta Online Server, with parameters set to use the starting coordinates in the ligand file and generate ligand conformers with the BCL. All other parameters were set to their default settings. Solutions from the top set of results were evaluated manually, and the final model was selected based on the absence of steric clashes and the ability to engage in substantial protein-ligand interactions.

### Cell culture methods

The human cell lines SH-SY5Y (SY5Y, ATCC CRL-2266), SK-MEL-5 (SKMEL-5, ATCC HTB-70), and NCI-H187 (H187, ATCC CRL-5804) were cultured in RPMI medium supplemented with 10% FBS and no antibiotics. These three cell lines were generously provided by the laboratory of Susan Lindquist via Luke Whitesell (currently at the University of Toronto, Toronto, ON, Canada). BJ-hTERT (described in [27], generously provided by Robert Weinberg, Whitehead Institute, Cambridge MA, USA) was cultured in 4:1 DMEM:M199 medium supplemented with 15% FBS and no antibiotics. All cell lines were grown and assayed at 37 °C, 5% CO_2_. All cell lines were screened for mycoplasma contamination by PCR (Mycoscope Detection Kit, Fisher Scientific) before use in assays.

For western blots, whole cell extracts were obtained by lysing cell culture pellets in 100 μL TNES buffer (50 mM Tris, 1% Igepal CA-630, 2 mM EDTA, 0.1 M NaCl) with Halt Protease Inhibitor Cocktail (Thermo Fisher Scientific) and centrifuging at maximum speed for 5 minutes to remove cellular debris. Supernatant containing 91 μg of total protein was diluted in gel loading buffer and boiled at 95 °C for 8 minutes. Samples were subjected to SDS-PAGE and standard Western Blotting. PDE5 was probed by 1:500 polyclonal rabbit anti-PDE5 (Sigma-Aldrich Cat# HPA004729, RRID:AB_1079588) and 1:5000 polyclonal goat horseradish peroxidase (HRP)-conjugated anti-rabbit (Sigma-Aldrich Cat# A0545, RRID:AB_257896). Tubulin served as a loading control and was probed by 1:2500 monoclonal mouse anti-tubulin (Abcam Cat# AB44928, RRID:AB_2241150) and 1:5000 polyclonal goat HRP-conjugated anti-mouse (Sigma-Aldrich Cat# AP308P, RRID:AB_92635).

For viability assays, cells were plated in black, clear bottom 384-well tissue culture-treated plates (Corning) and incubated overnight in the media described above but supplemented with 50 U/mL penicillin-streptomycin antibiotic (Life Technologies). Then, DMSO (no drug controls) and PDE5 inhibitors (0.01–10 μM; synthesized as described above or purchased from Selleck Chemicals) were added using a Hewlett-Packard D300 Digital Dispenser (Tecan) and incubated for 3 days. Maximum DMSO concentration was normalized to 0.1% of the total well volume for all wells. Resazurin (R & D Systems) was diluted 1:20 into each well and incubated for up to 4 hours. Fluorescence was read in a Tecan plate reader (excitation = 570 nm, emission = 585 nm). Wells treated with PDE5 inhibitors were normalized against control wells treated with DMSO to measure percent mitochondrial viability (100 = no effect on mitochondrial viability).

For assaying PDE5 inhibitor effects on GMP levels in a cell line, BJ-hTERT cells were plated in 6-well plates (Corning) in the media described above. When cells were 80% confluent, fresh, pre-warmed media with DMSO (untreated controls) or 20 μM sildenafil, **3**, or **7** was added and incubated for 1 hour. Maximum DMSO concentration in all samples was 0.2% of the total well volume. Cells were washed once with cold 0.9% NaCl. 800 μL of 80:20 methanol:water spiked with 500 nM isotopically labeled amino acids as internal standards was added while cells were on dry ice. Cells were detached with a cell scraper. Extracts were vortexed for 10 minutes and centrifuged at maximum speed at 4 °C to remove debris. Extracts were dried down in a vacuum dryer and stored at -80 °C. An additional sample was reserved for visual inspection of cell health by confocal light microscopy and for cell counting with a hemocytometer. Three independent assays were conducted in total, with 1–2 replicates per assay. To analyze GMP levels, samples were reconstituted in 50 μL LC-MS grade water and analyzed by HPLC-UV-MS as described in the general procedures and for enzyme inhibition and kinetics assays. The amino acid standards were analyzed in full scan positive mode with mass windows m/z 50–800. GMP peak areas were normalized to the peak area of the internal standard ^13^C4, ^15^N-threonine or ^13^C6, ^15^N3-histidine to account for variations in extraction or resuspension volume. The peak areas were then normalized to the average GMP peak area in undrugged controls and multiplied by 100 to obtain the percent GMP.

### Statistical analysis

All data are expressed as mean ± standard error of the mean (SEM). Statistical analysis was carried out using Student’s *t*-tests, ANOVA with Tukey’s multiple comparison test, or ANOVA with Dunnett’s multiple comparisons test as appropriate. All calculations were conducted using Prism 8. Default and recommended settings were used for all calculations.

## Supporting information

S1 Fig Characterization of compounds 1. (PDF)

S2 Fig Characterization of compounds 2. (PDF)

S3 Fig Characterization of compounds 3. (PDF)

S4 Fig Characterization of compounds 4. (PDF)

S5 Fig Characterization of compounds 5. (PDF)

S6 Fig Characterization of compounds 6. (PDF)

S7 Fig Characterization of compounds 7. (PDF)

S8 Fig Characterization of compounds 8. (PDF)

S9 Fig Characterization of compounds 9. (PDF)

S10 Fig Icariin and compounds 1–9 do not show characteristics of pan-assay interference compounds (PAINS). (A) Compounds were assayed for trypsin inhibition at 1x and 10x the PDE5 IC_50_ concentration. Compounds **3** and **7** were also assayed at 50x and 250x the IC_50_ concentration. Luteolin was assayed at 50, 100, and 250 μM (light grey, dark grey, and black outlined bars respectively). The addition of detergent is expected to reduce the non-specific inhibitory effects of PAINS compounds. The decrease in trypsin inhibition by luteolin in the presence of detergent further confirms luteolin as a PAINS compound. Values were measured in duplicate. Data is expressed as mean ± SEM. Average trypsin reaction velocity in no drug control reactions: 236 ± 2 nmol min^-1^ mg^-1^. *p<0.05, ***p<0.001 *vs* no drug controls (one-way ANOVA with Dunnet’s multiple comparisons test). (B) Icariin analogs at 1x the PDE5 IC_50_ concentration were assayed against a 2x increase in PDE5 concentration. Data was measured in triplicate and normalized to the untreated control median. Data is expressed as mean ± SEM. Average PDE5 reaction velocity in no drug control reactions: 1.50E-7 ± 4E-9 μmol/min with 6.25 ng PDE5; 3.4E-7 ± 1E-8 μmol/min with 12.5 ng PDE5. The difference between the mean of 6.25 ng PDE5 and the mean of 12.5 ng PDE5 was not statistically significant for any treatment (*t* test corrected for multiple comparisons with the Holm-Sidak method).
(PDF)

S11 Fig Icariin analog treatment shows minor effects on the viability of the four human cell lines SY5Y, SKMEL-5, BJ-hTERT, and NCI H187. A) Western blot showing PDE5 expression in SY5Y, SKMEL-5, BJ-hTERT, and NCI H187 human cell lines. Tubulin served as a loading control. (B-E) Effect of sildenafil or icariin analog treatment (0.01–10 μM) on the viability of four human cell lines as measured by resazurin. % viability was calculated by normalizing the resazurin fluorescence of drug treated cells to the resazurin fluorescence of untreated control cells. Data shown are mean ± SEM collected from three independent assays (SY5Y, SKMEL-5, and Bj-hTERT) or two independent assays (NCI H187) with 2–3 replicates per assay.
(PDF)

S12 Fig Original, uncropped Western blots of PDE5 (A) and tubulin (B) used in S11 Fig. S12-A and S12-B Figs were obtained as described in the Methods on a single gel that was transferred to a single nitrocellulose membrane and incubated in both anti-PDE5 and anti-tubulin antibodies. The membrane was cut in half in between the 75 and 50 kDa molecular weight marker to allow for different exposure times. S12-A was exposed for 1 minute, and S12-B was exposed for 5 minutes. Only the lanes for the cell lines SY5Y, SKMEL-5, BjHTERT, and NCI H187 (H187) were used to generate S11 Fig.
(PDF)

S13 Fig A representative GMP calibration curve measured using HPLC-UV-MS as described in the Methods section. GMP standard concentrations used were 5.9, 47, 375, 1500, and 3000 nM.
(PDF)

S14 Fig Dose-response curve and best-fit line as determined by nonlinear regression analysis for each icariin analog and sildenafil in *in vitro* PDE5 inhibition assays. Each data point represents the mean (from at least three replicates) ± SEM. Solid lines represent the best-fit line as determined by nonlinear regression analysis.
(PDF)

S15 Fig Original, untransformed Michaelis-Menten plots for PDE5 kinetics assays in the presence of compound 3 (A) or 7 (B). ● is no inhibitor, ○ is 0.015 μM inhibitor, ■ is 0.05 μM inhibitor, and □ is 0.2 μM inhibitor. Each data point represents the mean (from at least three replicates) ± SEM. Solid lines represent the best-fit line as determined by nonlinear regression analysis for a competitive inhibition model.
(PDF)

S1 Table PDE5 residues within hydrogen-bonding distance (<3.5 Å) to the icariin analog 7-*O* or 3-*O* functional group in ligand docking simulations. (PDF)

S2 Table Nonlinear regression analysis using Akaike’s Information Criteria to compare the fit between a competitive and noncompetitive inhibition model for inhibition of PDE5 enzyme by compound 3. (TXT)

S3 Table Nonlinear regression analysis using Akaike’s Information Criteria to compare the fit between a competitive and uncompetitive inhibition model for inhibition of PDE5 enzyme by compound 3. (TXT)

S4 Table Nonlinear regression analysis using Akaike’s Information Criteria to compare the fit between a competitive and noncompetitive inhibition model for inhibition of PDE5 enzyme by compound 7. (TXT)

S5 Table Nonlinear regression analysis using Akaike’s Information Criteria to compare the fit between a competitive and uncompetitive inhibition model for inhibition of PDE5 enzyme by compound 7. (TXT)

S1 Text file Modeling coordinates for compound 9 docked into human PDE5. Corresponds to Fig 4B.
(TXT)

S2 Text file Modeling coordinates for compound 3 docked into human PDE5. Corresponds to Fig 4C.
(TXT)
