# Supplementary material for: Exploration of icariin analog structure space reveals key features driving potent inhibition of human phosphodiesterase-5
Source: PLoS One. 2019 Sep 20;14(9):e0222803. doi: 10.1371/journal.pone.0222803 (PMC6754136; doi:10.1371/journal.pone.0222803)
Supplement: S2 Fig — (PDF) [file pone.0222803.s002.pdf]

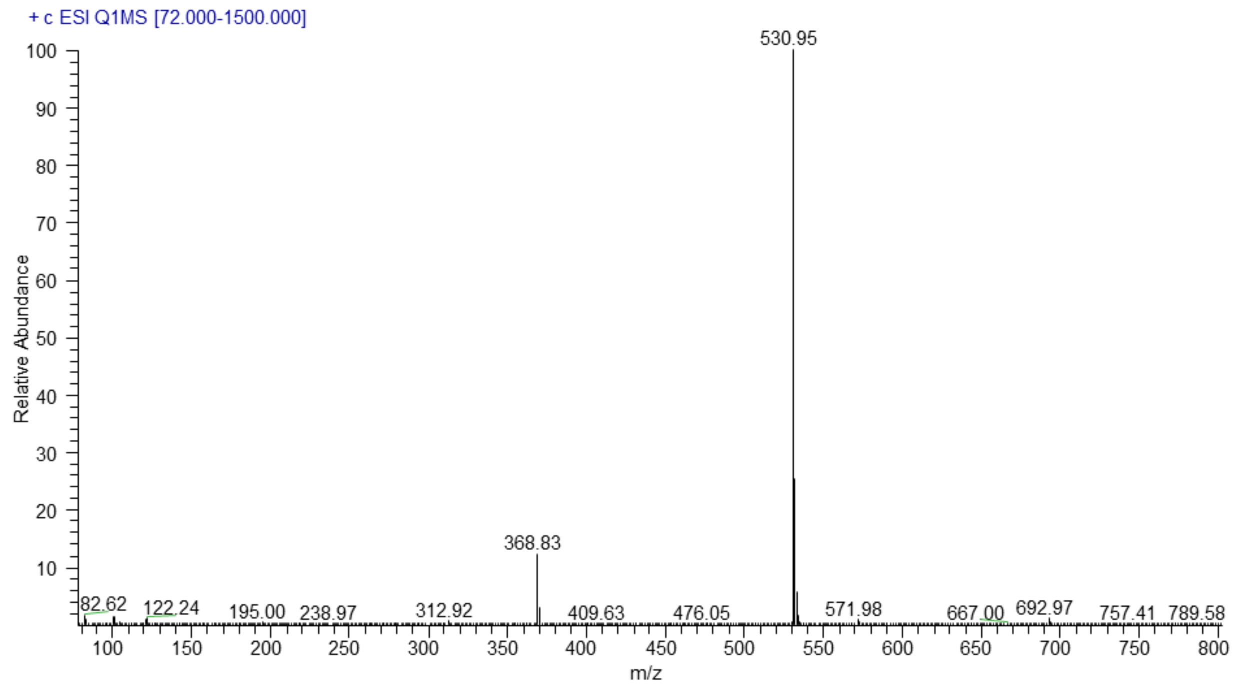


**S2-A Fig. Characterization of compound 2,** MS/MS spectrum of compound **2.**

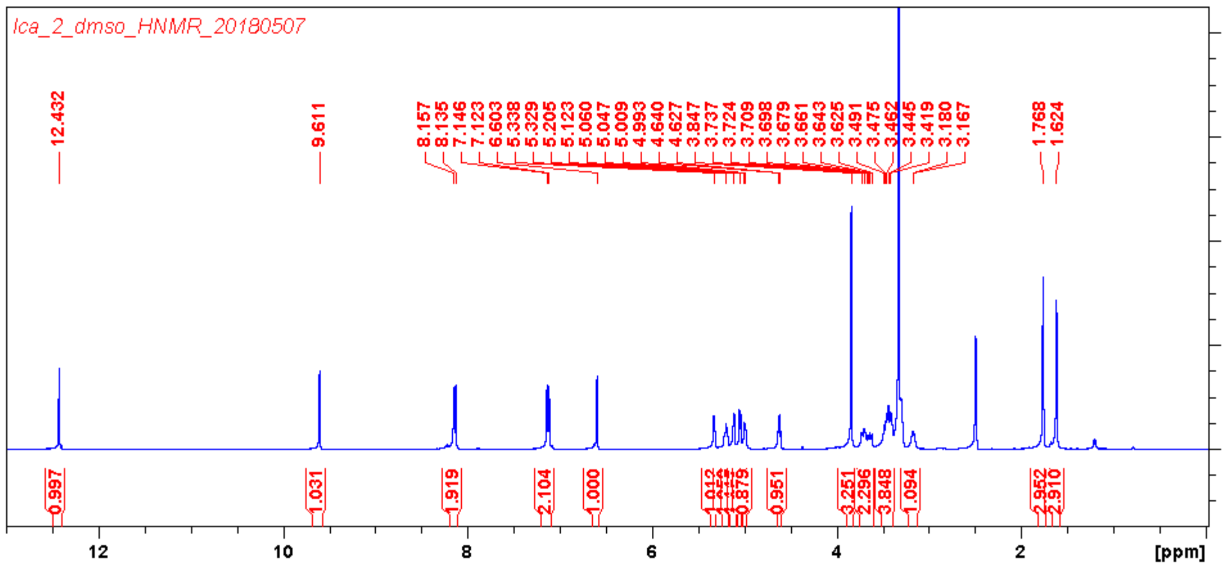


**S2-B Fig. Characterization of compound 2,** $^{1}$H NMR (400 MHz, DMSO-$d_6$) spectrum of compound **2.**

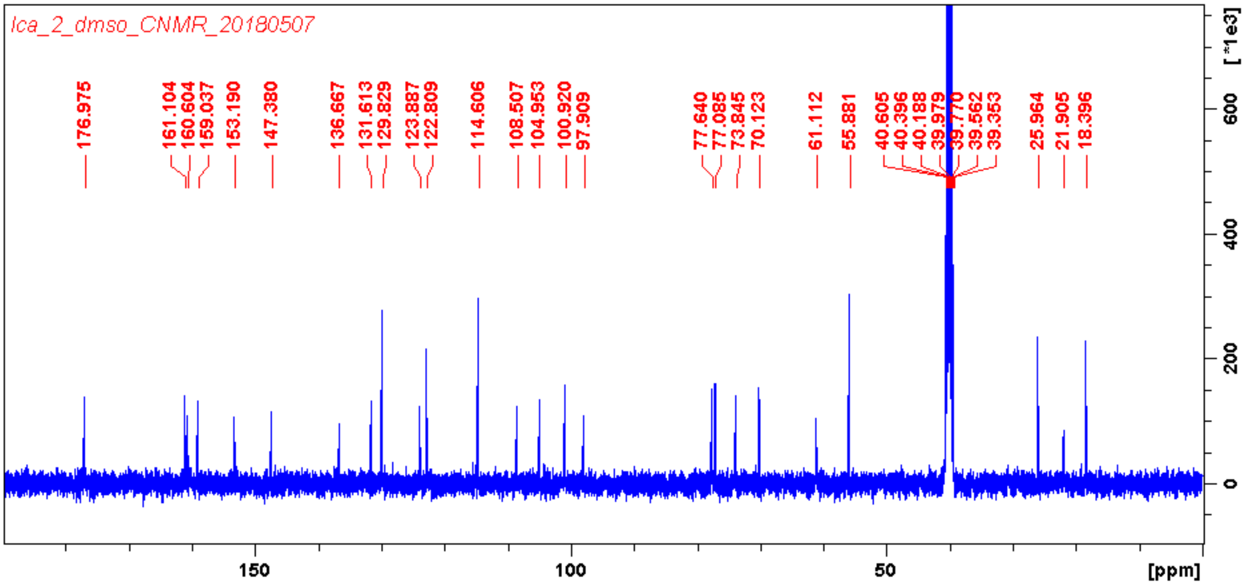


**S2-C Fig. Characterization of compound 2,** $^{13}C$ NMR (100 MHz, DMSO-$d_6$) spectrum of compound **2.**
