# Supplementary material for: Exploration of icariin analog structure space reveals key features driving potent inhibition of human phosphodiesterase-5
Source: PLoS One. 2019 Sep 20;14(9):e0222803. doi: 10.1371/journal.pone.0222803 (PMC6754136; doi:10.1371/journal.pone.0222803)
Supplement: S6 Fig — (PDF) [file pone.0222803.s006.pdf]

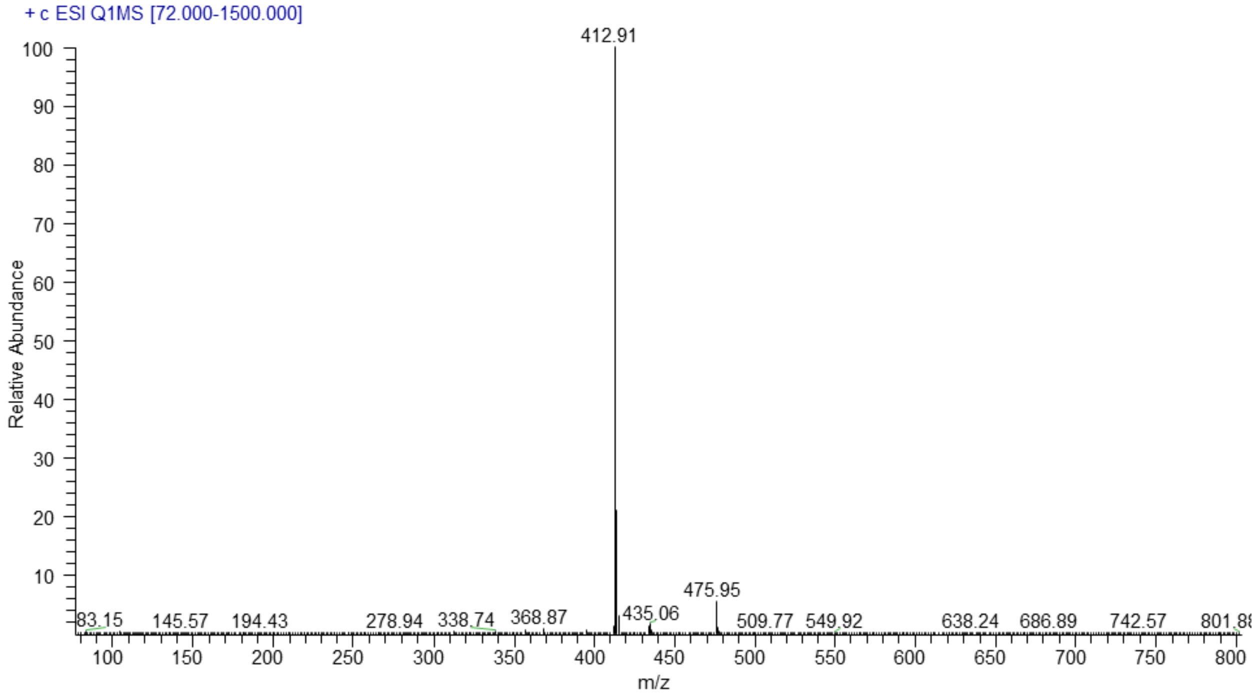

S6-A Fig. Characterization of compound 6, MS/MS spectrum of compound **6.**

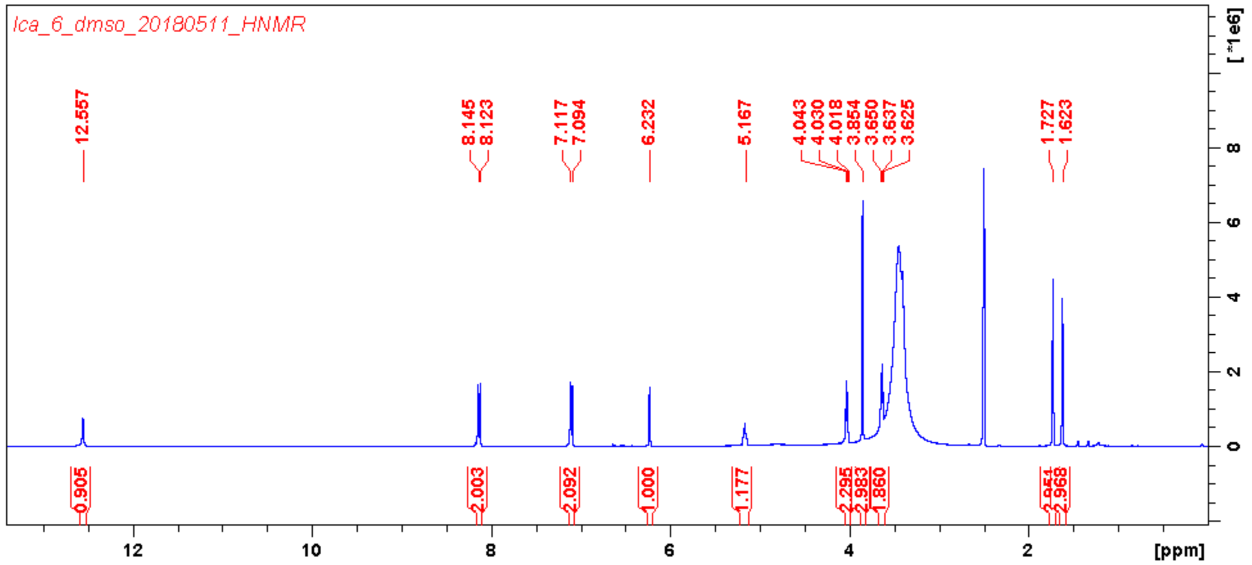

S6-B Fig. Characterization of compound 6, ^{1}H NMR (400 MHz, DMSO-d_6) spectrum of compound **6.**
