# Supplementary material for: Exploration of icariin analog structure space reveals key features driving potent inhibition of human phosphodiesterase-5
Source: PLoS One. 2019 Sep 20;14(9):e0222803. doi: 10.1371/journal.pone.0222803 (PMC6754136; doi:10.1371/journal.pone.0222803)
Supplement: S10 Fig — (A) Compounds were assayed for trypsin inhibition at 1x and 10x the PDE5 IC50 concentration. Compounds 3 and 7 were also assayed at 50x and 250x the IC50 concentration. Luteolin was assayed at 50, 100, and 250 μM (light grey, dark grey, and black outlined bars respectively). The addition of detergent is expected to reduce the non-specific inhibitory effects of PAINS compounds. The decrease in trypsin inhibition by luteolin in the presence of detergent further confirms luteolin as a PAINS compound. Values were measured in duplicate. Data is expressed as mean ± SEM. Average trypsin reaction velocity in no drug control reactions: 236 ± 2 nmol min-1 mg-1. *p<0.05, ***p<0.001 vs no drug controls (one-way ANOVA with Dunnet’s multiple comparisons test). (B) Icariin analogs at 1x the PDE5 IC50 concentration were assayed against a 2x increase in PDE5 concentration. Data was measured in triplicate and normalized to the untreated control median. Data is expressed as mean ± SEM. Average PDE5 reaction velocity in no drug control reactions: 1.50E-7 ± 4E-9 μmol/min with 6.25 ng PDE5; 3.4E-7 ± 1E-8 μmol/min with 12.5 ng PDE5. The difference between the mean of 6.25 ng PDE5 and the mean of 12.5 ng PDE5 was not statistically significant for any treatment (t test corrected for multiple comparisons with the Holm-Sidak method). (PDF) [file pone.0222803.s010.pdf]

A

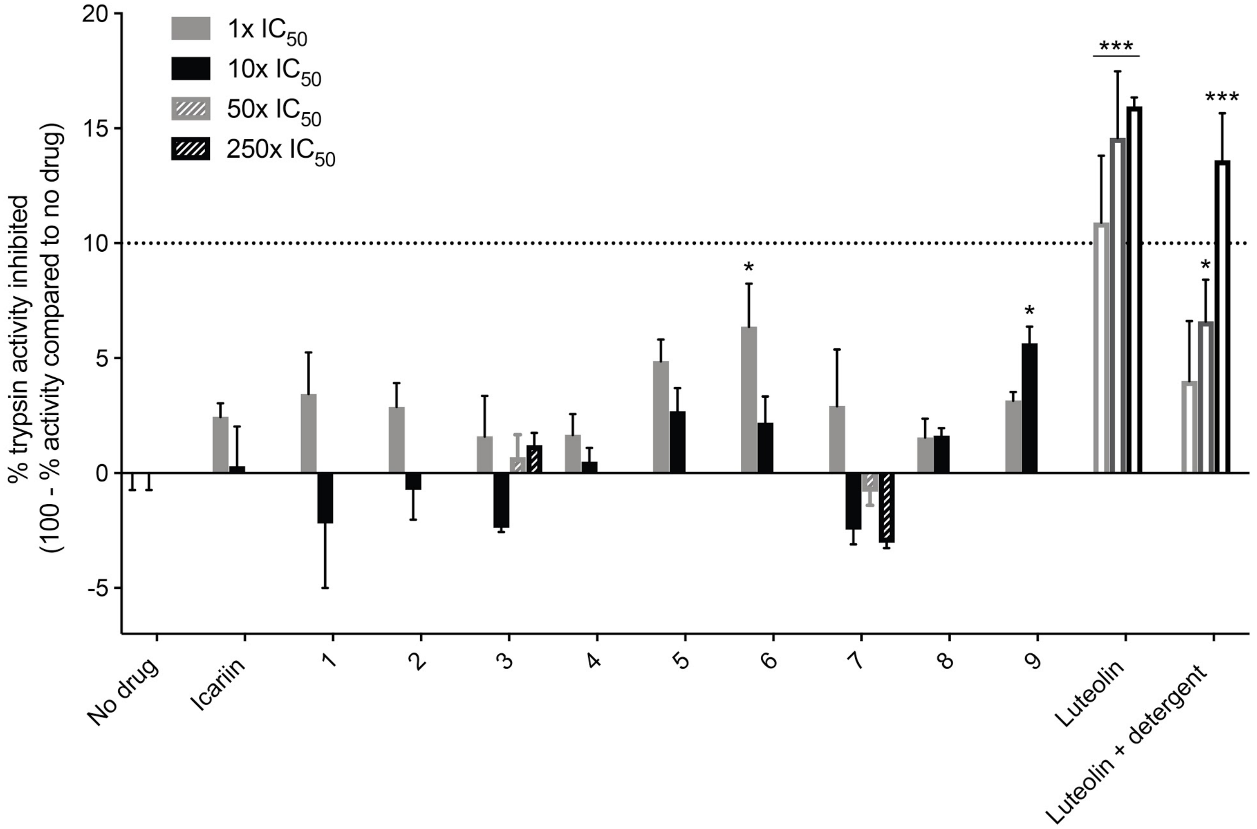

B

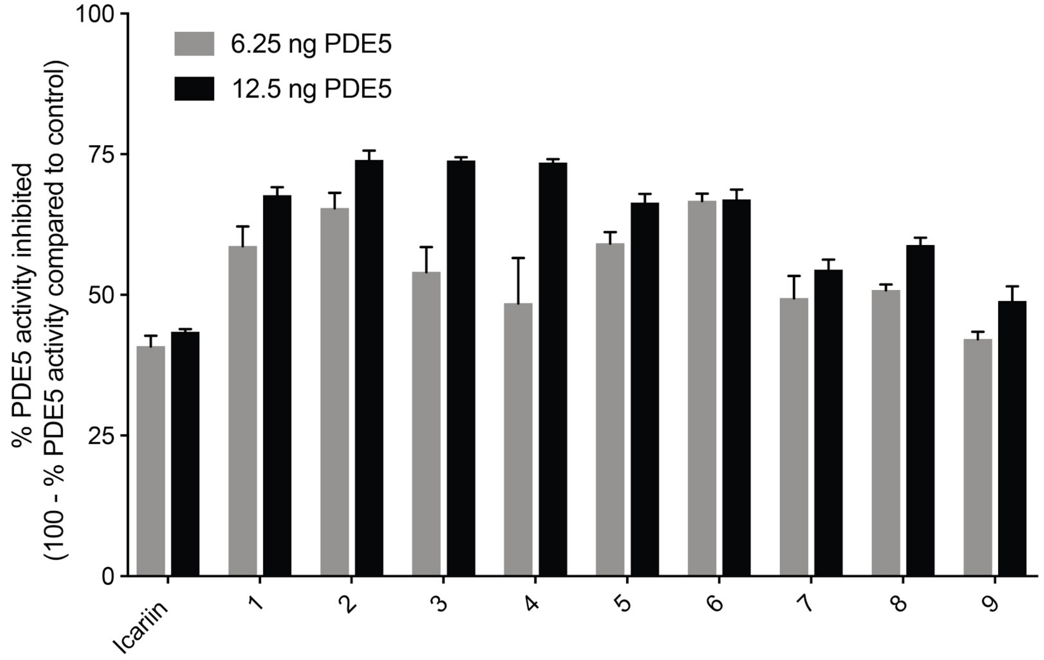

S10 Fig. Icariin and compounds 1-9 do not show characteristics of pan-assay interference compounds (PAINS). (A) Compounds were assayed for trypsin inhibition at 1x

and 10x the PDE5 IC_{50} concentration. Compounds **3** and **7** were also assayed at 50x and 250x the IC_{50} concentration. Luteolin was assayed at 50, 100, and 250 µM (light grey, dark grey, and black outlined bars respectively). The addition of detergent is expected to reduce the non-specific inhibitory effects of PAINS compounds. The decrease in trypsin inhibition by luteolin in the presence of detergent further confirms luteolin as a PAINS compound. Values were measured in duplicate. Data is expressed as mean ± SEM. Average trypsin reaction velocity in no drug control reactions: 236 ± 2 nmol min^{-1} mg^{-1}. *$p<0.05$, ***$p<0.001$ *vs* no drug controls (one-way ANOVA with Dunnet's multiple comparisons test). (B) Icariin analogs at 1x the PDE5 IC_{50} concentration were assayed against a 2x increase in PDE5 concentration. Data was measured in triplicate and normalized to the untreated control median. Data is expressed as mean ± SEM. Average PDE5 reaction velocity in no drug control reactions: 1.50E-7 ± 4E-9 µmol/min with 6.25 ng PDE5; 3.4E-7 ± 1E-8 µmol/min with 12.5 ng PDE5. The difference between the mean of 6.25 ng PDE5 and the mean of 12.5 ng PDE5 was not statistically significant for any treatment (*t* test corrected for multiple comparisons with the Holm-Sidak method). .
