# Supplementary material for: Exploration of icariin analog structure space reveals key features driving potent inhibition of human phosphodiesterase-5
Source: PLoS One. 2019 Sep 20;14(9):e0222803. doi: 10.1371/journal.pone.0222803 (PMC6754136; doi:10.1371/journal.pone.0222803)
Supplement: S11 Fig — A) Western blot showing PDE5 expression in SY5Y, SKMEL-5, BJ-hTERT, and NCI H187 human cell lines. Tubulin served as a loading control. (B-E) Effect of sildenafil or icariin analog treatment (0.01–10 μM) on the viability of four human cell lines as measured by resazurin. % viability was calculated by normalizing the resazurin fluorescence of drug treated cells to the resazurin fluorescence of untreated control cells. Data shown are mean ± SEM collected from three independent assays (SY5Y, SKMEL-5, and Bj-hTERT) or two independent assays (NCI H187) with 2–3 replicates per assay. (PDF) [file pone.0222803.s011.pdf]

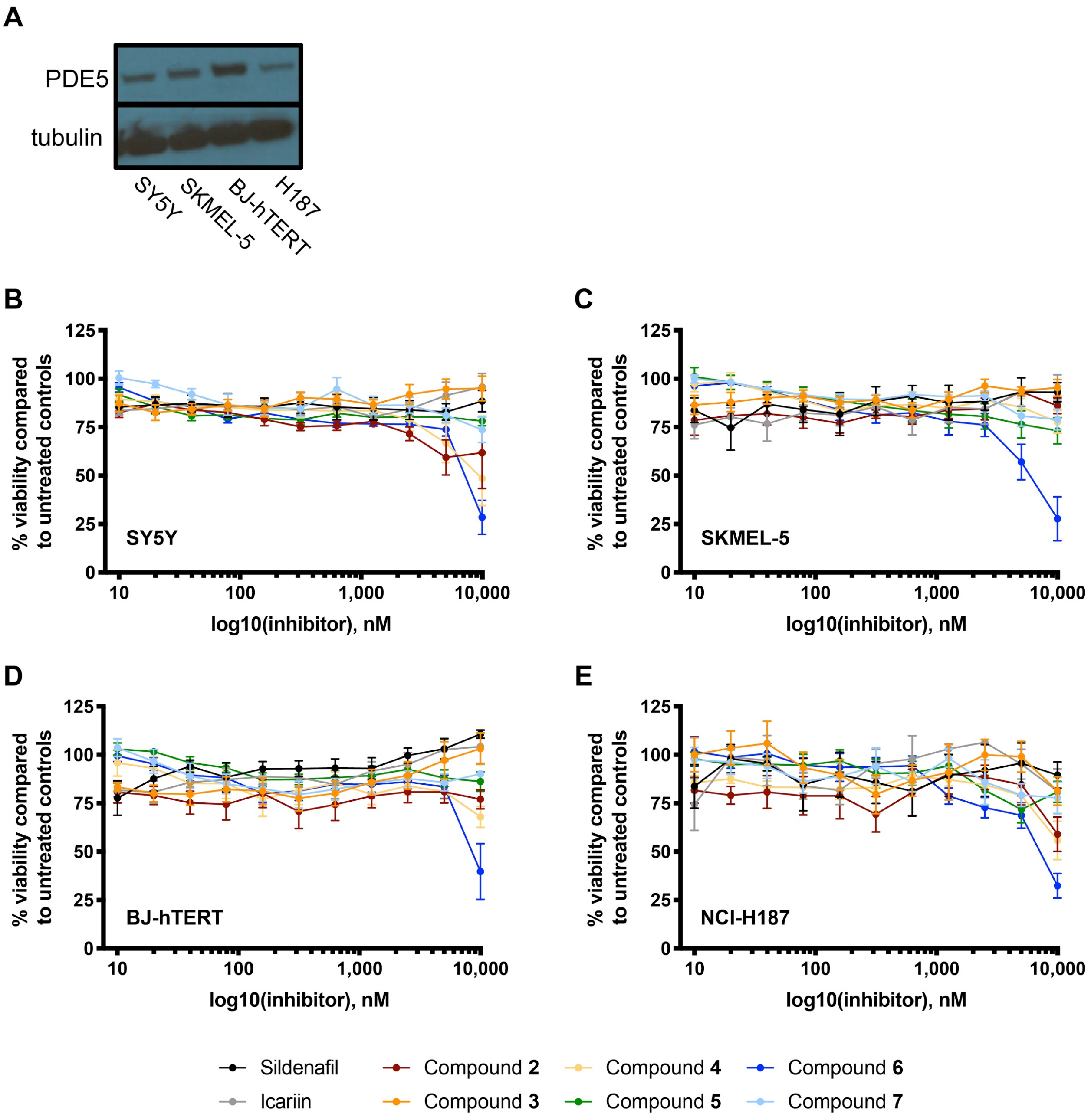

S11 Fig. Icariin analog treatment shows minor effects on the viability of the four human cell lines SY5Y, SKMEL-5, BJ-hTERT, and NCI H187. A) Western blot showing PDE5 expression in SY5Y, SKMEL-5, BJ-hTERT, and NCI H187 human cell lines. Tubulin served as a loading control. (B-E) Effect of sildenafil or icariin analog treatment (0.01-10 μM) on the viability of four human cell lines as measured by resazurin. % viability was calculated by normalizing the resazurin fluorescence of drug treated cells to the resazurin fluorescence of untreated control cells. Data shown are mean ± SEM collected from three independent assays (SY5Y, SKMEL-5, and Bj-hTERT) or two independent assays (NCI H187) with 2-3 replicates per assay.
