# Supplementary material for: Exploration of icariin analog structure space reveals key features driving potent inhibition of human phosphodiesterase-5
Source: PLoS One. 2019 Sep 20;14(9):e0222803. doi: 10.1371/journal.pone.0222803 (PMC6754136; doi:10.1371/journal.pone.0222803)
Supplement: S12 Fig — S12-A and S12-B Figs were obtained as described in the Methods on a single gel that was transferred to a single nitrocellulose membrane and incubated in both anti-PDE5 and anti-tubulin antibodies. The membrane was cut in half in between the 75 and 50 kDa molecular weight marker to allow for different exposure times. S12-A was exposed for 1 minute, and S12-B was exposed for 5 minutes. Only the lanes for the cell lines SY5Y, SKMEL-5, BjHTERT, and NCI H187 (H187) were used to generate S11 Fig. (PDF) [file pone.0222803.s012.pdf]

A

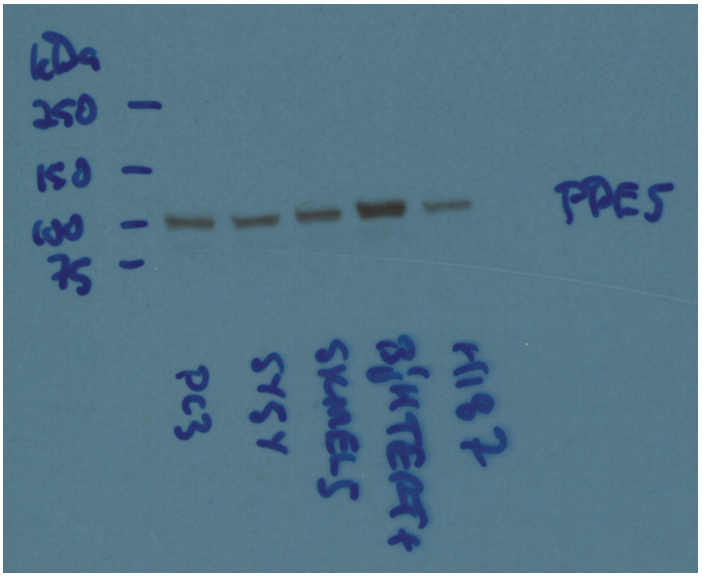

B

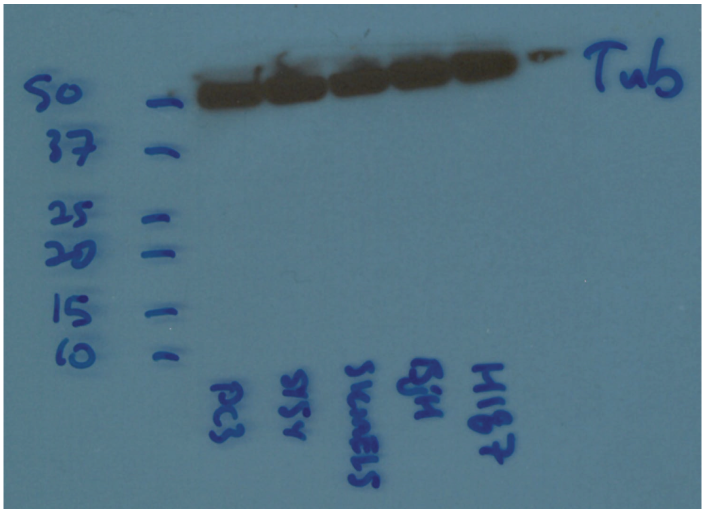

S12 Fig. Original, uncropped Western blots of PDE5 (A) and tubulin (B) used in Fig S11. Figs S12-A and S12-B were obtained as described in the Methods on a single gel that was transferred to a single nitrocellulose membrane and incubated in both anti-PDE5 and anti-tubulin antibodies. The membrane was cut in half in between the 75 and 50 kDa molecular weight marker to allow for different exposure times. S12-A was exposed for 1 minute, and S12-B was exposed for 5 minutes. Only the lanes for the cell lines SY5Y, SKMEL-5, BjHTERT, and NCI H187 (H187) were used to generate Fig S11.
