# Supplementary material for: Exploration of icariin analog structure space reveals key features driving potent inhibition of human phosphodiesterase-5
Source: PLoS One. 2019 Sep 20;14(9):e0222803. doi: 10.1371/journal.pone.0222803 (PMC6754136; doi:10.1371/journal.pone.0222803)
Supplement: S13 Fig — GMP standard concentrations used were 5.9, 47, 375, 1500, and 3000 nM. (PDF) [file pone.0222803.s013.pdf]

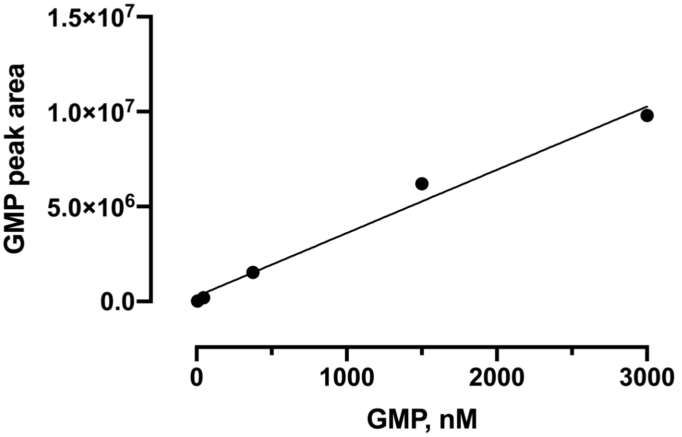

S13Fig. A representative GMP calibration curve measured using HPLC-UV-MS as described in the Methods section. GMP standard concentrations used were 5.9, 47, 375, 1500, and 3000 nM.
