# Supplementary material for: Exploration of icariin analog structure space reveals key features driving potent inhibition of human phosphodiesterase-5
Source: PLoS One. 2019 Sep 20;14(9):e0222803. doi: 10.1371/journal.pone.0222803 (PMC6754136; doi:10.1371/journal.pone.0222803)
Supplement: S14 Fig — Each data point represents the mean (from at least three replicates) ± SEM. Solid lines represent the best-fit line as determined by nonlinear regression analysis. (PDF) [file pone.0222803.s014.pdf]

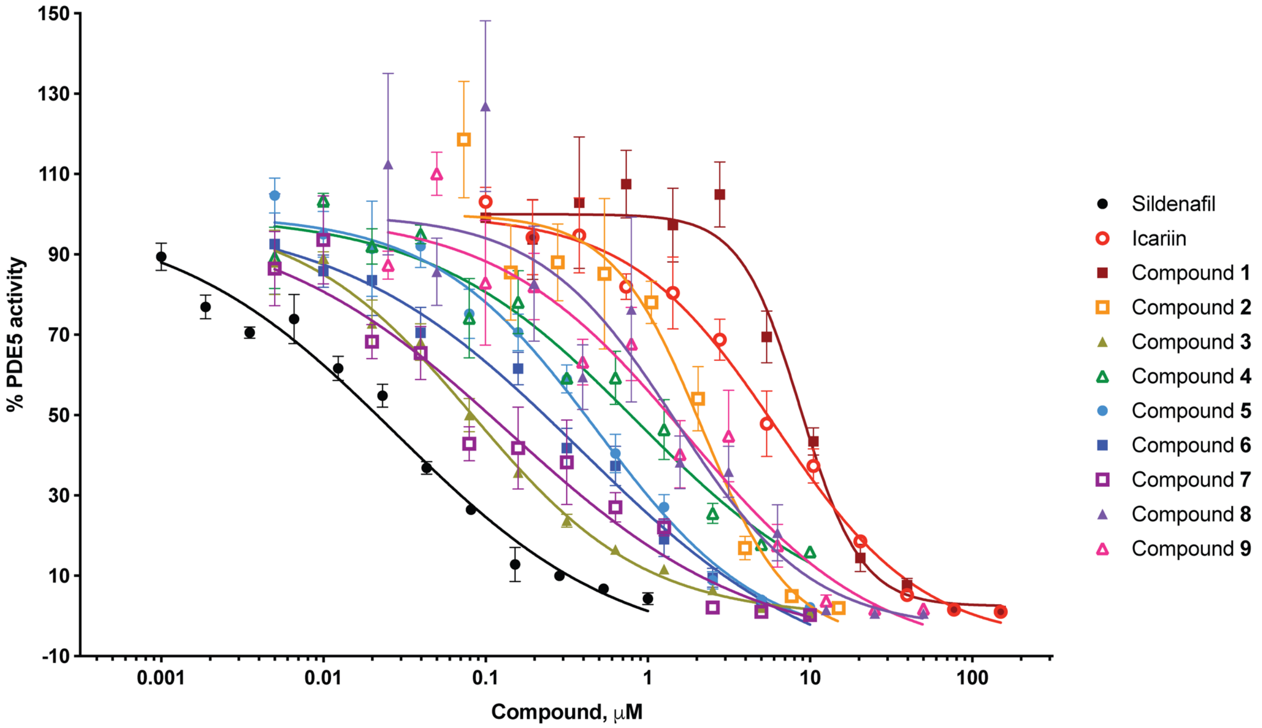

S14 Fig. Dose-response curve and best-fit line as determined by nonlinear regression analysis for each icariin analog and sildenafil in *in vitro* PDE5 inhibition assays. Each data point represents the mean (from at least three replicates) ± SEM. Solid lines represent the best-fit line as determined by nonlinear regression analysis.
