# Supplementary material for: Exploration of icariin analog structure space reveals key features driving potent inhibition of human phosphodiesterase-5
Source: PLoS One. 2019 Sep 20;14(9):e0222803. doi: 10.1371/journal.pone.0222803 (PMC6754136; doi:10.1371/journal.pone.0222803)
Supplement: S15 Fig — ● is no inhibitor, ○ is 0.015 μM inhibitor, ■ is 0.05 μM inhibitor, and □ is 0.2 μM inhibitor. Each data point represents the mean (from at least three replicates) ± SEM. Solid lines represent the best-fit line as determined by nonlinear regression analysis for a competitive inhibition model. (PDF) [file pone.0222803.s015.pdf]

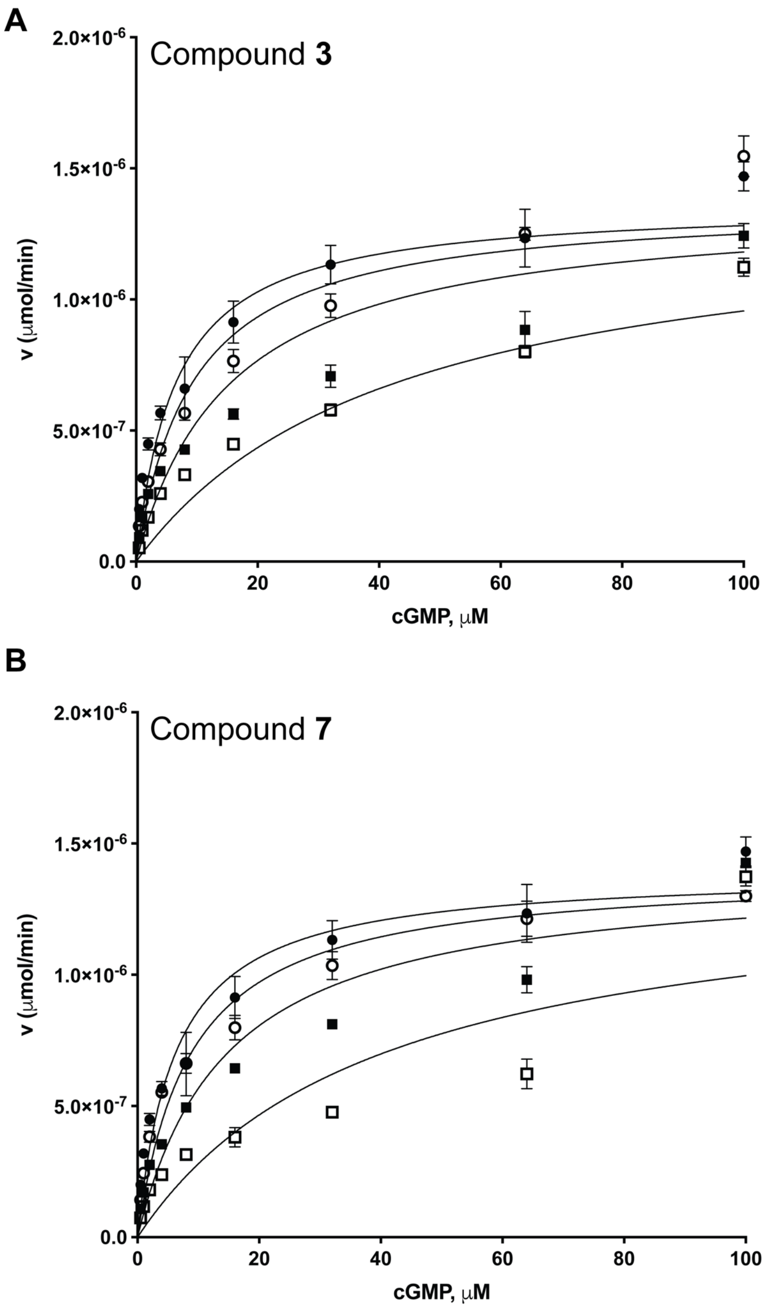

S15 Fig. Original, untransformed Michaelis-Menten plots for PDE5 kinetics assays in the presence of compound 3 (A) or 7 (B). ● is no inhibitor, ○ is 0.015 μM inhibitor, ■ is 0.05 μM inhibitor, and □ is 0.2 μM inhibitor. Each data point represents the mean (from at least three replicates) ± SEM. Solid lines represent the best-fit line as determined by nonlinear regression analysis for a competitive inhibition model.
