# Supplementary material for: Exploration of icariin analog structure space reveals key features driving potent inhibition of human phosphodiesterase-5
Source: PLoS One. 2019 Sep 20;14(9):e0222803. doi: 10.1371/journal.pone.0222803 (PMC6754136; doi:10.1371/journal.pone.0222803)
Supplement: S1 Table — (PDF) [file pone.0222803.s016.pdf]

S1 Table. PDE5 residues within hydrogen-bonding distance (<3.5Å) to the icariin analog 7-*O* or 3-*O* functional group in ligand docking simulations

	7-*O* position	3-*O* position
Icariin	Ser668 (x2), Arg667, Ser668, Glu669, Leu804(x2)	His613
1 (icariside II)	Ser668	Asp764, His613
2[a]		
3	Ile665, Arg667, Ser668 (x2), Glu669, Leu804	Asp764
4	Ser668, Ile665, Arg667, Phe820 (main chain)	Leu725
5	Ser668 (x3), Leu804	Asp764, Leu765
6	Ser668	Asp764
7	Ser668	Asp764, Thr723, Leu725
8	Ser668	His613
9	Ser668	

[a]A reasonable model could not be determined
